# Identification of WRKY gene family members in amaranth based on a transcriptome database and functional analysis of *AtrWRKY42-2* in betalain metabolism

**DOI:** 10.3389/fpls.2023.1300522

**Published:** 2023-12-05

**Authors:** Rongzhi Yang, Tianliu Huang, Weiwei Song, Zixian An, Zhongxiong Lai, Shengcai Liu

**Affiliations:** Institute of Horticultural Biotechnology, Fujian Agriculture and Forestry University, Fuzhou, Fujian, China

**Keywords:** *Amaranthus tricolor* L., betalain, *AtrWRKY42-2* gene, relative expression level of WRKY TFs, functional analysis

## Abstract

**Introduction:**

WRKY TFs (WRKY transcription factors) contribute to the synthesis of secondary metabolites in plants. Betalains are natural pigments that do not coexist with anthocyanins within the same plant. *Amaranthus tricolor* (‘Suxian No.1’) is an important leaf vegetable rich in betalains. However, the WRKY family members in amaranth and their roles in betalain synthesis and metabolism are still unclear.

**Methods:**

To elucidate the molecular characteristics of the amaranth WRKY gene family and its role in betalain synthesis, WRKY gene family members were screened and identified using amaranth transcriptome data, and their physicochemical properties, conserved domains, phylogenetic relationships, and conserved motifs were analyzed using bioinformatics methods.

**Results:**

In total, 72 WRKY family members were identified from the amaranth transcriptome. Three WRKY genes involved in betalain synthesis were screened in the phylogenetic analysis of WRKY TFs. RT-qPCR showed that the expression levels of these three genes in red amaranth ‘Suxian No.1’ were higher than those in green amaranth ‘Suxian No.2’ and also showed that the expression level of *AtrWRKY42* gene short-spliced transcript *AtrWRKY42-2* in Amaranth ‘Suxian No.1’ was higher than that of the complete sequence *AtrWRKY42-1*, so the short-spliced transcript *AtrWRKY42-2* was mainly expressed in ‘Suxian No.2’ amaranth. Moreover, the total expression levels of *AtrWRKY42-1* and *AtrWRKY42-2* were down-regulated after GA_3_ treatment, so *AtrWRKY42-2* was identified as a candidate gene. Therefore, the short splice variant *AtrWRKY42-2* cDNA sequence, gDNA sequence, and promoter sequence of *AtrWRKY42* were cloned, and the PRI 101-AN-*AtrWRKY42-2*-EGFP vector was constructed to evaluate subcellular localization, revealing that *AtrWRKY42-2* is located in the nucleus. The overexpression vector pRI 101-AN-*AtrWRKY42-2*-EGFP and VIGS (virus-induced gene silencing) vector pTRV2-*AtrWRKY42-2* were transferred into leaves of ‘Suxian No.1’ by an *Agrobacterium*-mediated method. The results showed that *AtrWRKY42-2* overexpression could promote the expression of *AtrCYP76AD1* and increase betalain synthesis. A yeast one-hybrid assay demonstrated that *AtrWRKY42-2* could bind to the *AtrCYP76AD1* promoter to regulate betalain synthesis.

**Discussion:**

This study lays a foundation for further exploring the function of *AtrWRKY42-2* in betalain metabolism.

## 1 Introduction

Amaranth (*Amaranthus tricolor* L.) is an annual plant with beneficial health effects. It is cultivated in many countries. It is native to India (Parveen et al., 2018) and distributed in southern Asia (Wang et al., 2019), Central Asia, Japan, and other places. Amaranth is rich in betalains, characteristic pigments in Amaranthaceae (Chang et al., 2021), and betalain structure in the species differs from that of betalain in beet. Amaranth is an important resource for betalain extraction and research (Zheng et al., 2016).

Betalains exist in only 15 families in Caryophyllales and the fungus *Amanita muscaria* (Clement and Mabry, 1996; Polturak et al., 2018). The stability of betalains from different sources varies in the temperature range of 50–90°C; accordingly, botanists use the thermal stability of betacyanins as a chemical classification index (Martins et al., 2017; Carreón-Hidalgo et al., 2022). Betalains are classified as betacyanins and betaxanthins. There are four main types of betacyanins: amaranthin, betanin, gomphrenin, and decarboxybetanin (Strack et al., 2003; Cai et al., 2006). Amaranthin is biosynthesized from betanin (Gins et al., 2002) and has only been detected in the genus *Amaranthus*. The betalain synthesis pathway in plants has been characterized. Cytochrome P450 enzyme CYP76AD catalyzes tyrosine to l-DOPA (Polturak et al., 2016), Subsequently, l-DOPA is catalyzed to an open cyclo-DOPA by 4,5-dopa dioxygenase extradiol (or DODA enzyme) and then spontaneously forms betalamic acid, which is the core skeleton of all betalain compounds and can spontaneously combine with amino acids or amines to produce betaxanthin (Polturak and Aharoni, 2018; Sheehan et al., 2020). At the same time, dopamine is oxidized by the cytochrome P450 enzyme CYP76AD1 to form cyclo-DOPA, and cyclo-DOPA spontaneously condenses to form beta-glucoside ligand, which then generates betacyanin under the action of beta-glucoside ligand-5-*O*-glucosyl transferase (Hatlestad et al., 2012; Hatlestad et al., 2015; Alfonso et al., 2019). MYB transcription factors regulate the synthesis of betalain via *DODA* and *CYP76AD1* (Xie et al., 2016; Peng et al., 2019). In addition, WRKY TFs in pitaya participate in the regulation of *CYP76AD1* and thus regulate betalain synthesis (Cheng et al., 2017; Zhang et al., 2021).

WRKYs are a plant-specific transcription factor family, named for the conserved WRKYGQK7 amino acid sequence. This family has been identified in *Arabidopsis* (Birkenbihl et al., 2018), sorghum (Baillo et al., 2020), maize (Hu et al., 2021), pitaya (Chen et al., 2022), *Beta vulgaris* (Cheng et al., 2017; Wu et al., 2019; Zhang et al., 2021), banana (Jia et al., 2022), and other taxa. WRKY proteins could specifically bind to the TTGAC sequence (also known as W-box) to regulate gene transcription, and their expression is mainly induced by pathogenic bacteria, injury, and SA (salicylic acid) (Li et al., 2020). WRKY proteins are involved in plant development, metabolic regulation (Zhang et al., 2018; Su et al., 2022), plant stress responses (Li et al., 2020), and aging . WRKY TFs regulate betalain metabolism in pitaya. In particular, *HmoWRKY40*, *HpWRKY44*, *HpWRKY3*, and *HpWRKY18* play important roles in the color transformation and betalain accumulation of pitaya (Cheng, 2018; Zhang, 2020).

We have identified genes involved in betalain metabolism in amaranth, such as *AtrCYP76AD1*, *AtrDODA*, *AtrDOPA5-GT*, *AtrB5-GT*, and *AtrB6-GT*, by transcriptomics analyses (Zheng et al., 2016). However, the specific WRKY TFs in amaranth involved in betalain synthesis and the underlying regulatory mechanisms have not been determined. In this study, we identified WRKY TFs and demonstrated that *AtrWRKY42-2* interacts with the promoter of *AtrCYP76AD1*, thereby regulating the betalain synthesis in amaranth. These findings provide new insight into betalain synthesis.

## 2 Materials and methods

### 2.1 Materials and treatment

**Figure 1:**
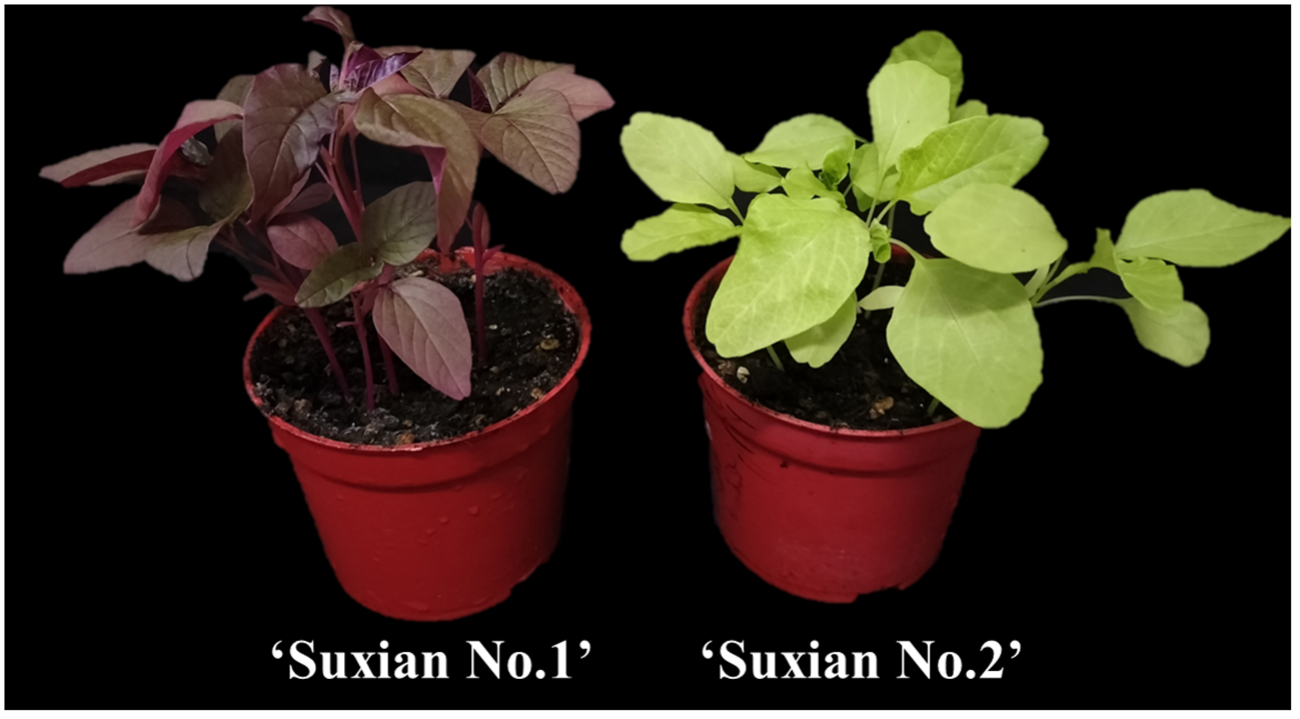
Phenotypic differences between typical potted seedlings of ‘Suxian No.1’ (left) and ‘Suxian No.2’ (right).

Seeds of ‘Suxian No.1’ and ‘Suxian No.2’ were provided by Suzhou Academy of Agricultural Sciences. ‘Suxian No.1’ was dark red and rich in betalains. ‘Suxian No.2’ was light green and did not contain betalains (**Figure 1**). The leaves (functional leaves) of ‘Suxian No.1’ from potted seedlings at about one month were used for transient transformation and virus-induced gene silencing (VIGS). All samples were frozen in liquid nitrogen immediately and stored at −80°C for further analyses. Briefly, 1 mg/L GA_3_ and 2 mg/L paclobutrazol (PP_333_) were added into MS medium supplemented with sucrose (30 g/L) and agar (7 g/L) (Pan et al., 2018; Zhao et al., 2019; Xiao et al., 2021). The sterilized seeds of ‘Suxian No.1’ and ‘Suxian No.2’ were inoculated in the medium. Medium without hormones was used as a control. Each treatment was evaluated with 15 bottles and three biological replicates. After 7 days of culture, the hypocotyl at the amaranth seedling stage was collected.

### 2.2 Identification of *WRKY* gene family members in amaranth

#### 2.2.1 Data sources, gene identification, and physical parameters

Transcriptome data for amaranth were obtained from our constructed database (SRA:SRX7816306–SRX7816311) (Liu et al., 2019). Gene screening was performed using hidden Markov models (HMM). The WRKY domain was downloaded from Pfam (PF03106) (http://pfam.xfam.org/, accessed on October 19, 2021) and was employed to identify all possible WRKY genes in amaranth using HMMER (Chen et al., 2015) with an e-value threshold of -5. Each candidate WRKY gene was further confirmed using SMART (http://smart.embl-heidelberg.de/) and the Conserved Domain Database (CDD, http://www.ncbi.nlm.nih.gov/Structure/cdd/wrpsb.cgi). Physical parameters, including the molecular mass and theoretical isoelectric point (pI) of the deduced WRKY proteins were investigated using ExPASy (http://web.expasy.org/protparam/).

#### 2.2.2 Analyses of conserved motifs and conserved domains of WRKY proteins

Conserved motifs of AtrWRKY proteins were identified using MEME (Multiple Expectation Maximization for Motif Elicitation) [http://meme-suite.org/tools/meme (accessed on October 29, 2022)].

Amino acid sequence logos of WRKY were generated using the WebLogo platform (https://weblogo.berkeley.edu/logo.cgi). DNAMAN7 (company: Lynnon Corporation; Version 7.0.2.176) was used to compare the amino acid sequences of WRKY homologies in different species.

#### 2.2.3 Phylogenetic analysis of *AtrWRKY*s

Full-length amino acid sequences for a phylogenetic analysis were obtained from pitaya (Cheng et al., 2017; Chen et al., 2022) and *Beta vulgaris* (Wu et al., 2019). A phylogenetic tree was constructed by the neighbor-joining method (NJ) using MEGA11 (Version 11.0.8) with 1000 boot-strap replicates. Evolview (https://www.evolgenius.info/evolview/#/treeview) was used to visualize the phylogenetic tree.

#### 2.2.4 RT-qPCR analyses

Total RNA was isolated from samples using MolPure Plant Plus RNA Kit (Yeasen, China) according to the manufacturer's instructions. First-strand cDNA was synthesized from 1 μg of total RNA using Recombinant M-MLV reverse transcriptase (TransGen Biotech, Beijing, China). Quantitative real time-PCR (qRT-PCR) was performed in optical 96-well plates using the Roche LightCycler 480 instrument (Roche, Sweden). The reactions were carried out in a 20 μL volume containing 10 μL of SYBR Premix Ex Taq, 0.8 μL of gene-specific primers, 2 μL f diluted cDNA, and 6.4 μL of ddH_2_O. The PCR conditions were as follows: 30 s at 95°C, 45 cycles of 10 s at 95°C and 20 s at 59°C, followed by 12 s at 72°C. Three biological repeats were performed for each material. *SAND* (Xiao et al., 2021) was used as the internal reference gene. Excel 2016 was used for data analyses, SPSS 20 was used to evaluate the homogeneity of variances, and the 2^−ΔΔCt^ method was used for quantitative analyses of gene expression. The primer pairs used for the qRT-PCR analysis of *WRKY* genes are listed in **Supplementary Table S1**.

### 2.3 Cloning and subcellular localization

#### 2.3.1 Cloning and sequence analysis of *AtrWRKY42-2*

The cDNA and gDNA sequences of *AtrWRKY42-2* were cloned from ‘Suxian No.1’ and ‘Suxian No.2’ samples. The genomic sequence and its corresponding coding sequences (CDS) were compared using GSDS (Gene Structure Display Server) (http://gsds.cbi.pku.edu.cn/).

#### 2.3.2 Subcellular localization of *AtrWRKY42-2*

The full-length coding sequence of *AtrWRKY42-2* was inserted into the pRI 101-AN-EGFP vector (primers are listed in **Supplementary Table S1**). *Agrobacterium* strain GV3101 cells carrying pRI 101-AN-*AtrWRKY42*-*2*-EGFP and pRI101-AN-EGFP were infiltrated into onion inner epidermis cells. Transient expression of EGFP was recorded using a laser confocal microscope (Olympus model: FV1200 4 laser) at 405 nm. Onion epidermis cells were stained with DAPI to evaluate nuclear localization, and the fluorescence signal was visualized by laser confocal microscopy at 473 nm.

#### 2.3.3 Promoter cloning and sequence analysis

The promoter of *AtrWRKY42-2* was cloned from total genomic DNA of ‘Suxian No.1’ using the TaKaRa Genome Walking Kit (see **Supplemental Table S1** for specific primers). Promoter elements and conserved binding domains of *AtrWRKY42-2* were analyzed using PlantCARE (http://bioinformatics.psb.ugent.be/webtools/plantcare/html/, accessed on November 10, 2022).

### 2.4 Functional analysis of *AtrWRKY42-2*

pRI 101-AN-*AtrWRKY42-2*-EGFP and pRI 101-AN-EGFP were transformed into the *Agrobacterium tumefaciens* strain GV3101. The bacterial cells were resuspended to an OD600 of 0.8–1.0 using MMA buffer (10 mM MES, 10 mM MgCl_2_, 100 μM acetosyringone) at a 1:1 ratio. Subsequently, bacterial cells were infiltrated into amaranth leaves. Then these seedlings were cultured in the dark at 25°C for 1–2 days. These seedlings were then transferred to a culture room set to 16 h/8 h (light/dark) and 25°C. Plant phenotypes were observed. The transiently transformed plants were cultured for 1 week (including dark treatment for 2 days), and the 7th to 8th true leaves were collected to detect gene expression levels and determine the betalain content.

The gene fragment with the *AtrWRKY42-2* conserved domain was cloned into the pTRV2 vector. pTRV1, pTRV2, and pTRV2-*AtrWRKY42-2* were transformed into *Agrobacterium tumefaciens* strain GV3101. The bacterial cells were resuspended to an OD600 of 0.6~0.8 using an MMA buffer. pTRV2 (negative control) and pTRV2- *AtrWRKY42-2* were separately infiltrated into amaranth leaves with pTRV1 in a ratio of 1:1. The culture conditions were the same as those described above. The leaves were collected for the detection of gene expression level and the determination of betalain contents.

### 2.5 Measurement of betalain contents

Betalain contents were determined as described by Hua et al. (Hua et al., 2016) The absorption peaks of betacyanin and betaxanthin at 538 nm and 465 nm were determined by ultraviolet-visible spectrum spectrophotometer (UV-900, Shanghai Yuan Analysis Instrument Co., Ltd.). The betacyanin and betaxanthin contents were calculated according to a molar extinction coefficient of betaxanthin of 5.66 × 10^4^. All determinations were performed in triplicate.

### 2.6 Yeast one-hybrid assay of *AtrWRKY42-2* and the *AtrCYP76AD1* promoter

The promoter of *AtrCYP76AD1* (see **Supplementary Text S2** for the sequence) was ligated to the pHis II yeast reporter vector to form a pPromoter-pHis II recombinant bait vector. *AtrWRKY42-2* was ligated into the expression vector pGADT7 to construct the pGADT7-AD recombinant vector and the pGADT7-*AtrWRKY42-2* vector plasmid was used to transform the positively verified *AtrCYP76AD1*- pHis II (primers are listed in **Supplementary Table S1**). The bait yeast strain Y187 was cultured at 30°C for 3 days. Two deficient media, SD/-Leu-Trp and SD/-Leu-Trp-His-Ade, were used to detect the interaction between *AtrWRKY42-2* and the *AtrCYP76AD1* promoter based on yeast growth.

## 3 Results

### 3.1 Identification and physical parameters of WRKY gene family members in amaranth

A total of 72 putative WRKY genes were screened by a systematic analysis of the transcriptome of *A. tricolor*. These WRKY genes were termed *AtrWRKY1* to *AtrWRKY72.* The full-length CDS of the *AtrWRKY* genes ranged from 818 bp (*AtrWRKY12* and *AtrWRKY31*) to 5459 bp (*AtrWRKY15*) with deduced proteins of 178–1093 amino acids. Furthermore, the computed molecular weights of these WRKY proteins ranged from 19517.74 to 124261.7 Da. The theoretical pI of the deduced *AtrWRKY* proteins ranged from 5.11 to 9.96. The detailed sequences and annotation are listed in **Supplementary Table S3**.

### 3.2 Classification and phylogenetic tree analysis of Amaranth AtrWRKYs

#### 3.2.1 Conserved motifs and domains of AtrWRKYs

**Figure 2:**
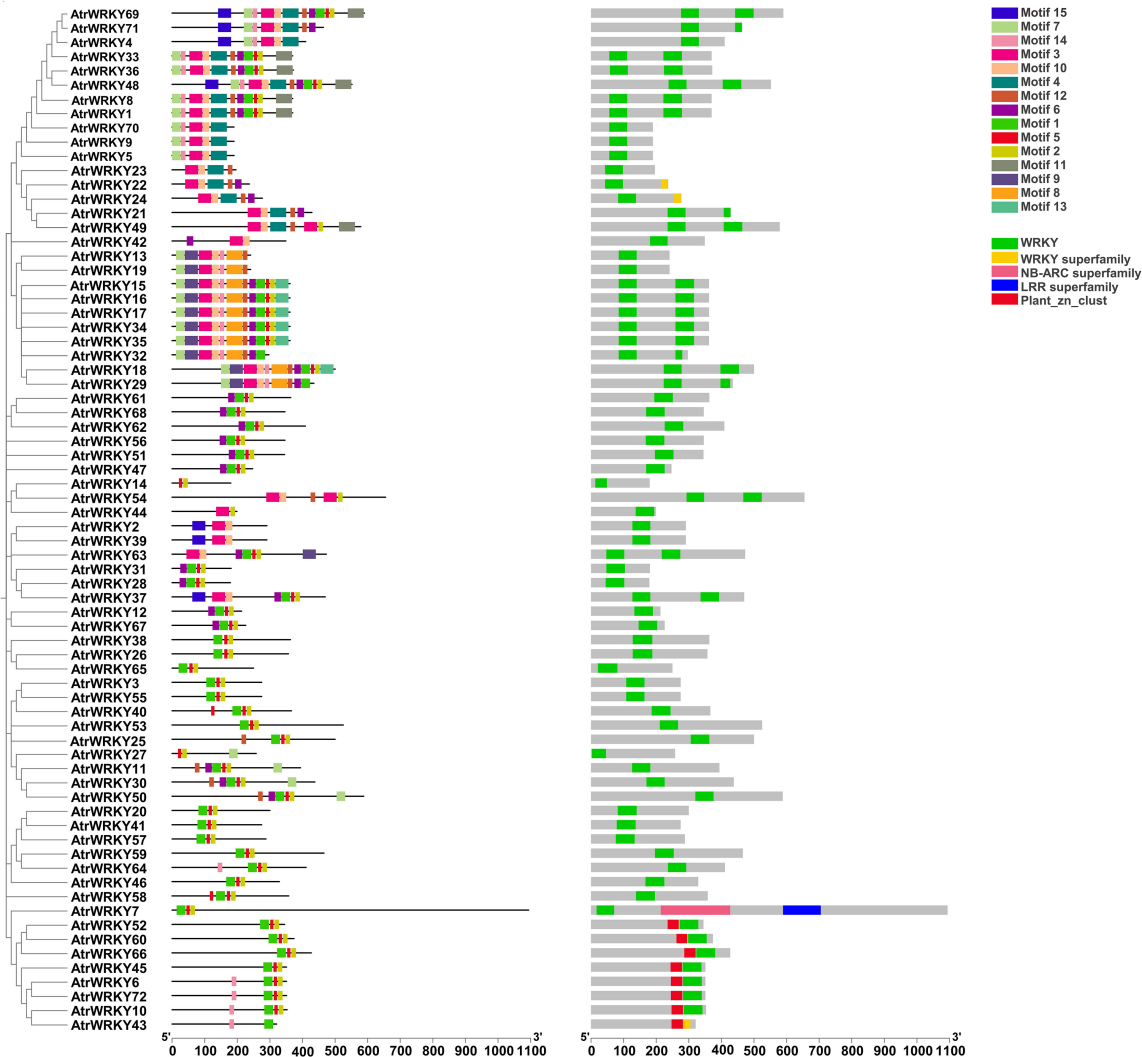
Analysis of conserved motifs and conserved domains of AtrWRKYs. Motif1–15 represents the conserved sequence of AtrWRKYs members. WRKY and WRKY superfamily represent the conservative domain of AtrWRKYs members.LRR superfamily; NB-ARC superfamily represents the LRR and NB-ARC domains; Plant_zn_clust represent plant zinc cluster.(AtrWRKYs: Amaranth WRKY transcription factor family).

A total of 15 conserved motifs were detected from AtrWRKYs using the MEME tool. Each motif contained 15–50 amino acids, and each sequence included 2–12 motifs. Motifs 1 and 3 formed the main structure of the WRKY domain (WRKYGQK), and Motifs 2, 10, and 13 contained a zinc-finger motif. The most frequent motifs of AtrWRKYs were Motif 1, Motif 5, and Motif 2, which showed high amino acid sequence conservation and represent the AtrWRKY domain (**Figure 2**). *AtrWRKY6*, *AtrWRKY72*, *AtrWRKY10*, and *AtrWRKY43* all had Motif 14. However, Motif 9 was only identified in *AtrWRKY63*.

#### 3.2.2 Phylogenetic analysis and structural classification of WRKY genes in *A. tricolor*

**Figure 3:**
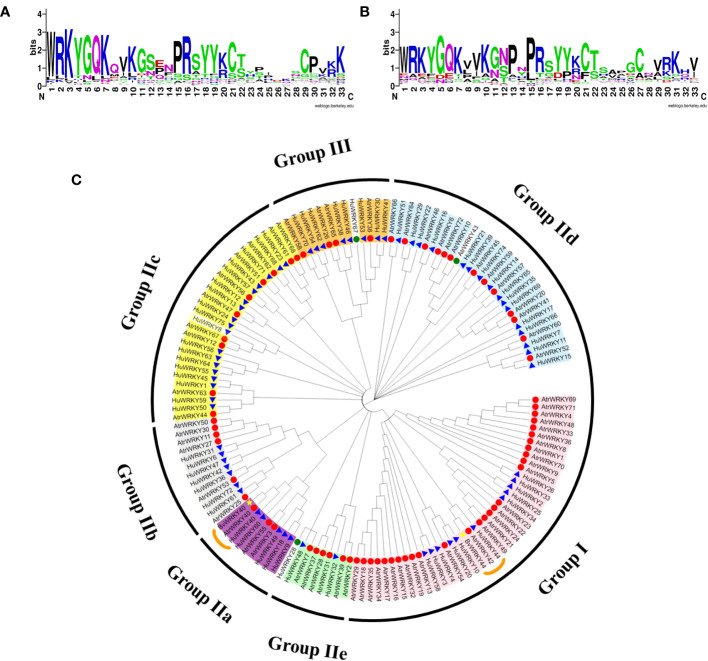
Conserved amino acid sequence analysis and WRKY phylogenetic tree analysis. **(A)** Comparison of the conserved domain (WRKY-a) of AtrWRKY family members; **(B)** Comparison of the WRKY-b conserved domain (The higher the bit value in the log map of the domain, the more conserved the corresponding amino acid; WRKY-a: upstream domain of the gene sequence; WRKY-b: downstream domain of gene sequence); **(C)** WRKY phylogenetic tree (Red circle represents amaranth, blue triangle represents pitaya, and yellow star represents sugar beet. The green circle represents the WRKY members classified in group IV).

At the amino acid sequence level, the family motifs were well conserved (**Figures 3A, B**). To evaluate the evolutionary history of the *WRKY* gene family in *A. tricolor* and to uncover their classification and function in betalain synthesis, 72 WRKY proteins from *A. tricolor*, *Beta vulgaris* (betalain), and ‘Guan Huahong’pitaya (betalain) were used to construct a phylogenetic tree using the NJ method implemented in MEGA11. Among the selected AtrWRKY7, AtrWRKY40, and *AtrWRKY42-2* proteins, WRKY homologs in the same group/subgroup as those in sugar beet and pitaya may have similar functions. *AtrWRKY7* and *AtrWRKY40* were homologous to *AtWRKY25* and *HmoWRKY40*, respectively. In particular, *AtrWRKY42-2* was homologous to *HpWRKY44*, *AtWRKY44*, and *BvWRKY44*.We predicted that *AtrWRKY7*, *AtrWRKY40*, and *AtrWRKY42-2* might be involved in betalain synthesis (**Figure 3C**).

An un-rooted phylogenetic tree was constructed based on the alignments of the WRKY motif and zinc-finger motif from amaranth using the NJ method (**Figures 2**, **3C**). The AtrWRKYs were divided into four groups (Groups I–IV). Group I contained two WRKY domains and C_2_H_2_ type zinc finger protein domains, including 19 AtrWRKYs. Group II contained one WRKY domain and C_2_H_2_ type zinc finger protein domain, including 48 AtrWRKYs. Group III only contained one zinc finger motif of type C_2_HC and one protein domain (Huang et al., 2012; Wang et al., 2014), including four AtrWRKYs (*AtrWRKY65*, *AtrWRKY38*, *AtrWRKY58*, and *AtrWRKY26*). *AtrWRKY43* belonged to Group IV, which contained one WRKY domain and an incomplete C_2_H_2_ type zinc finger protein domain (Eulgem et al., 2000).

### 3.3 Sequence comparison of transcript *AtrWRKY42-1* and *AtrWRKY42-2*

**Figure 4:**
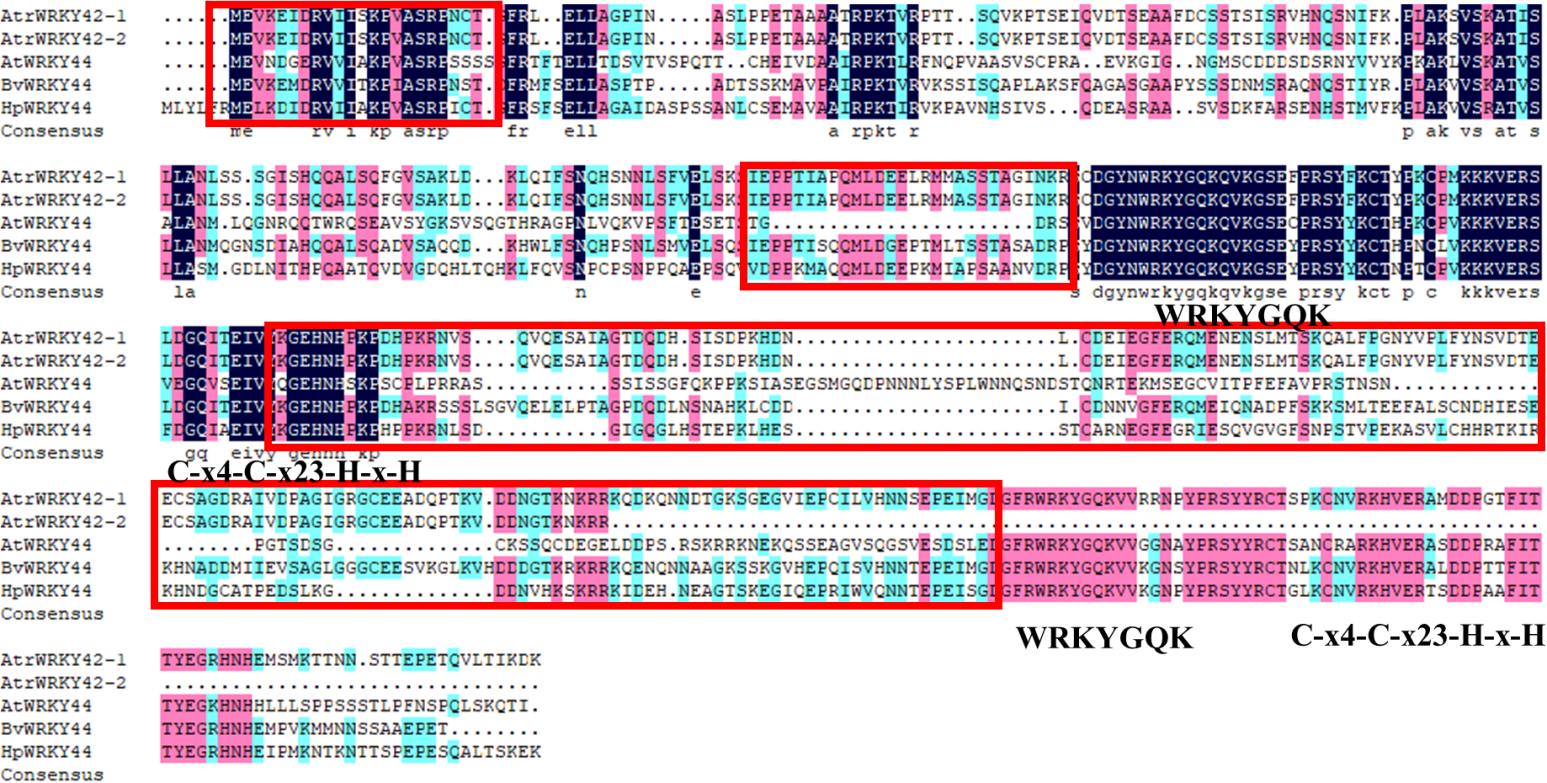
Homologous gene protein difference. WRKYGQK: WRKY domain; Zinc finger structure: C-x4-C-x23-H-x-H; Red box is the betalain synthesis-related WRKY specific amino acid sequence. “Atr” stands for Amaranth, “At” stands for *Arabidopsis*, “Bv” stands for *B. vulgaris*, and “Hp” stands pitaya.

As shown in **Figure 4**, the complete transcript sequence was named *AtrWRKY42-1* (CL2645.Contig1_All), and short splice transcript was one of the transcripts of the *AtrWRKY42* gene, named *AtrWRKY42-2* (CL2645.Contig2_All). *AtrWRKY42-2* had only one conserved domain, which was one fewer domain than the complete protein-coding gene, and it could be confirmed as a member of Group II according to the WRKY classification criteria.

A sequence alignment of *WRKY44* homologues in beet, pitaya, and *Arabidopsis* (see **Supplementary Text S4** for sequence details) showed that the other four proteins were clearly different from the *AtWRKY44* protein motif (**Figure 4**). We further analyzed the *AtrWRKY42-2* conserved domains. WRKY proteins involved in betalain synthesis from pitaya, *B. vulgaris, Arabidopsis*, and amaranth revealed that *AtrWRKY42-2* had the same domain and zinc finger structure as those of the *WRKY44* cluster. Except for *AtWRKY44*, the other three genes had special amino acid sequence fragments (**Figure 4**).

### 3.4 Expression patterns under treatment with plant growth regulators

**Figure 5:**
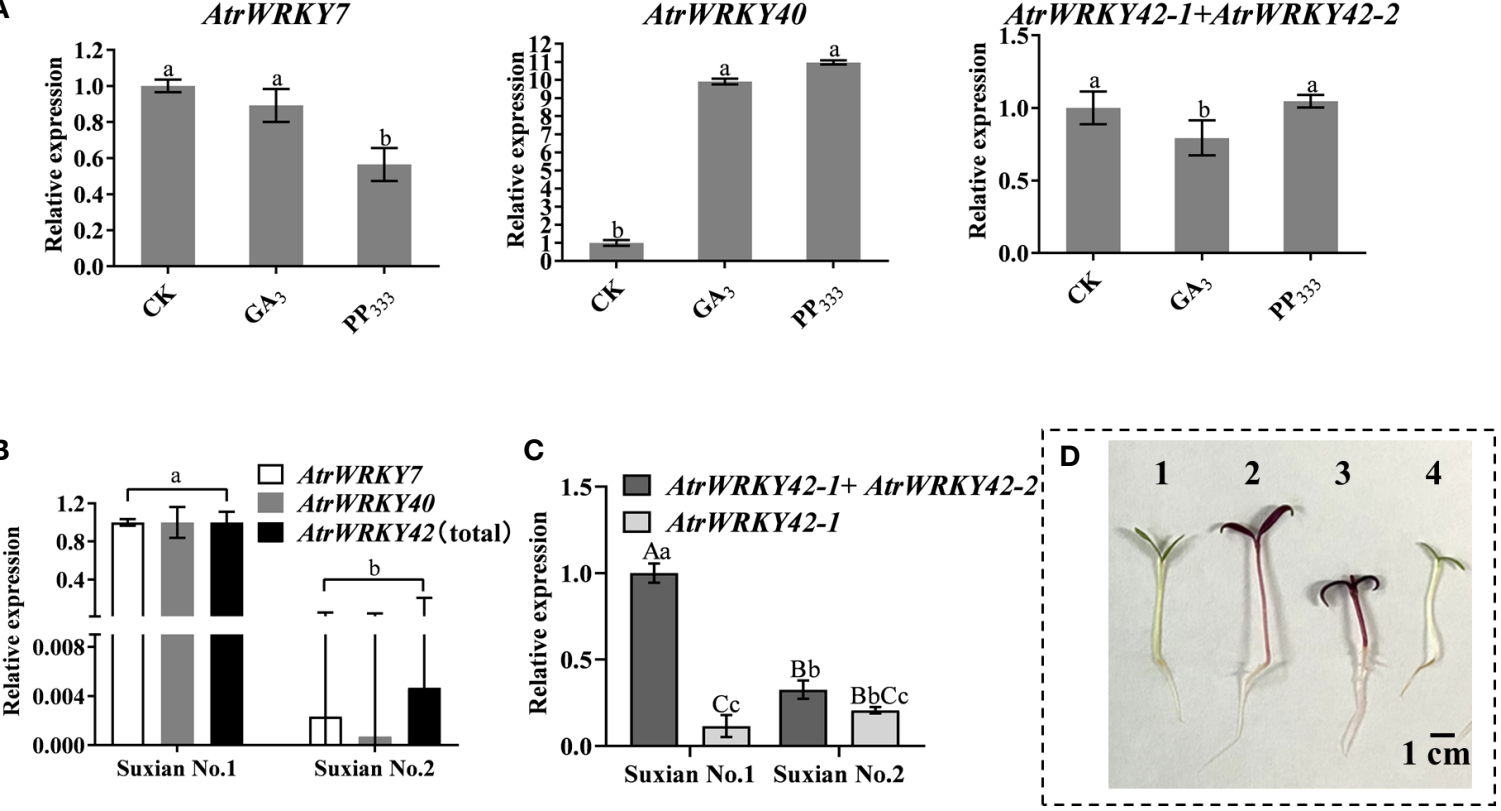
Quantitative analysis of *AtrWRKY7*, *AtrWRKY40*, and *AtrWRKY42* expression in different varieties and after hormone treatment. **(A)** Quantitative expression analysis under different hormone treatments; **(B)** Quantitative expression in different under different varieties; **(C)**. The relative expression levels of *AtrWRKY42-1* and *AtrWRKY42-2* in two varieties; **(D)** Plant phenotypes 1–3 indicate the seedlings of ‘Suxian No.1’ treated with GA_3_, sterile water, and PP_333_, respectively, and 4 indicates seedlings of ‘Suxian No.2’ treated with sterile water. (CK: ‘Suxian No.1’ blank control; GA_3_: Optimal concentration of 1 mg/L; PP_333_: Optimal concentration of 2 mg/L; *AtrWRKY42*(total): It represents the total expression of *AtrWRKY42-1* and *AtrWRKY42-2*; Unprocessed ‘Suxian No.1’ material (CK) was used for calibration (set as 1) in three independent experiments (n = 3), and the error bar represents twice the standard error. The difference was statistically significant when compared with levels in CK by Student’s *t*-tests; a, b indicate a significant difference at p < 0.05; Aa, Bb and Cc indicate a significant difference at p < 0.01.

To further identify betalain-related *WRKY* genes from *A. tricolor*, the expression patterns of *AtrWRKY7\40\42* genes in the leaves of ‘Suxian No.1’ and ‘Suxian No.2’ were determined by RNA-seq. We have previously found that GA_3_, 2,4-D, and 6-BA promote the growth of amaranth, the accumulation of betacene in amaranth is inhibited by GA_3_ and 2,4-D, and PP_333_ promotes the synthesis of betacene (Liu et al., 2018; Pan et al., 2018). Therefore, we determined the expression patterns of *AtrWRKY7\40\42* genes under treatment with 1 mg/L GA_3_ and 2 mg/L paclobutrazol to verify the expression of the putative WRKY TFs. As shown in **Figure 4**, the expression levels of *AtrWRKY7\40\42* were altered in the ‘Suxian No.1’ leaves. In particular, *AtrWRKY40* was upregulated under GA_3_ treatment, *AtrWRKY42-2* was downregulated, and *AtrWRKY7* expression did not differ between treatment and control groups. *AtrWRKY40* was upregulated under paclobutrazol treatment, *AtrWRKY7* was downregulated, and *AtrWRKY42* was upregulated (**Figure 5**). Based on these results, we selected *AtrWRKY42* as a candidate gene for further analyses. However, the total expression levels of *AtrWRKY42-1* and *AtrWRKY42-2* in ‘Suxian No.1’ and’Suxian No.2’ were always higher than the expression levels of *AtrWRKY42-1*, so the short splicing transcript *AtrWRKY42-2* expressed mainly in the plants of the two varieties.

### 3.5 Sequence analysis of *AtrWRKY42-2*

#### 3.5.1 Sequence features

**Figure 6:**
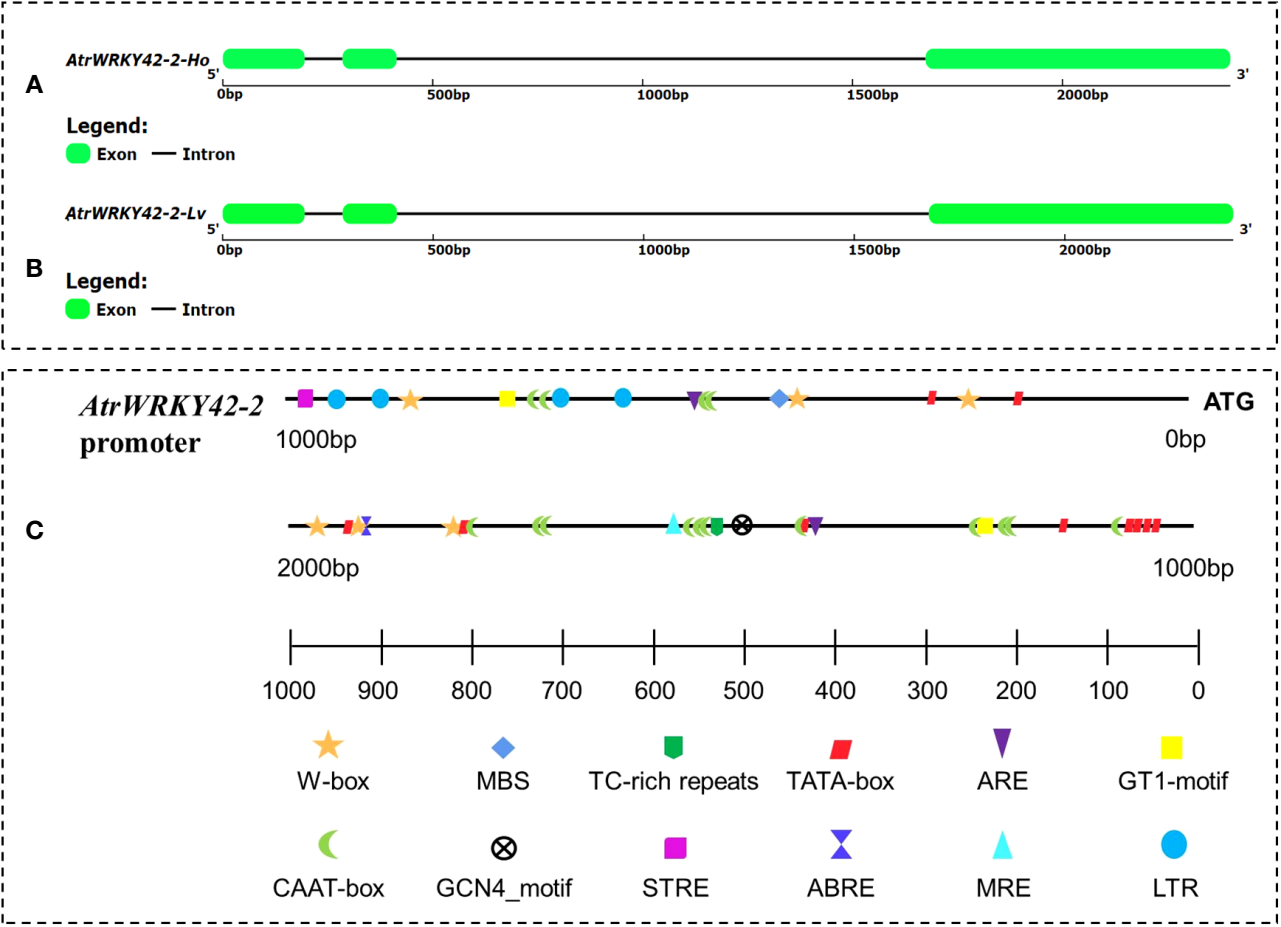
*AtrWRKY42-2* gene structure and *AtrWRKY42-2* cis-regulatory elements. **(A)** Gene structure of *AtrWRKY42-2* in ‘Suxian No.1’ variety; **(B)** Gene structure of *AtrWRKY42-2* in ‘Suxian No.2’ variety; **(C)** Distribution of cis-acting elements of the *AtrWRKY42-2* promoter.

Complementary DNA and genomic DNA of *AtrWRKY42-2* were cloned from both ‘Suxian No.1’ and ‘Suxian No.2’. There was no difference in the cDNA sequence of *AtrWRKY42-2* between ‘Suxian No.1’ and ‘Suxian No.2’, encoding 348 amino acids, with an isoelectric point (pI) of 7.58 (see **Supplementary Table S3**), and the gDNA sequences were identical (**Figures 6A, B**).

A 1891 bp promoter region (see **Supplementary Text S2** for the sequence) was successfully amplified. In this region, a transcription start site (TSS), a TATA-box 1000 bp upstream of the TSS (**Figure 6C**), and a large number of other cis-acting elements (**Supplementary Table S5**) were predicted based on a PlantCARE database analysis.

#### 3.5.2 Subcellular localization of *AtrWRKY42-2*

**Figure 7:**
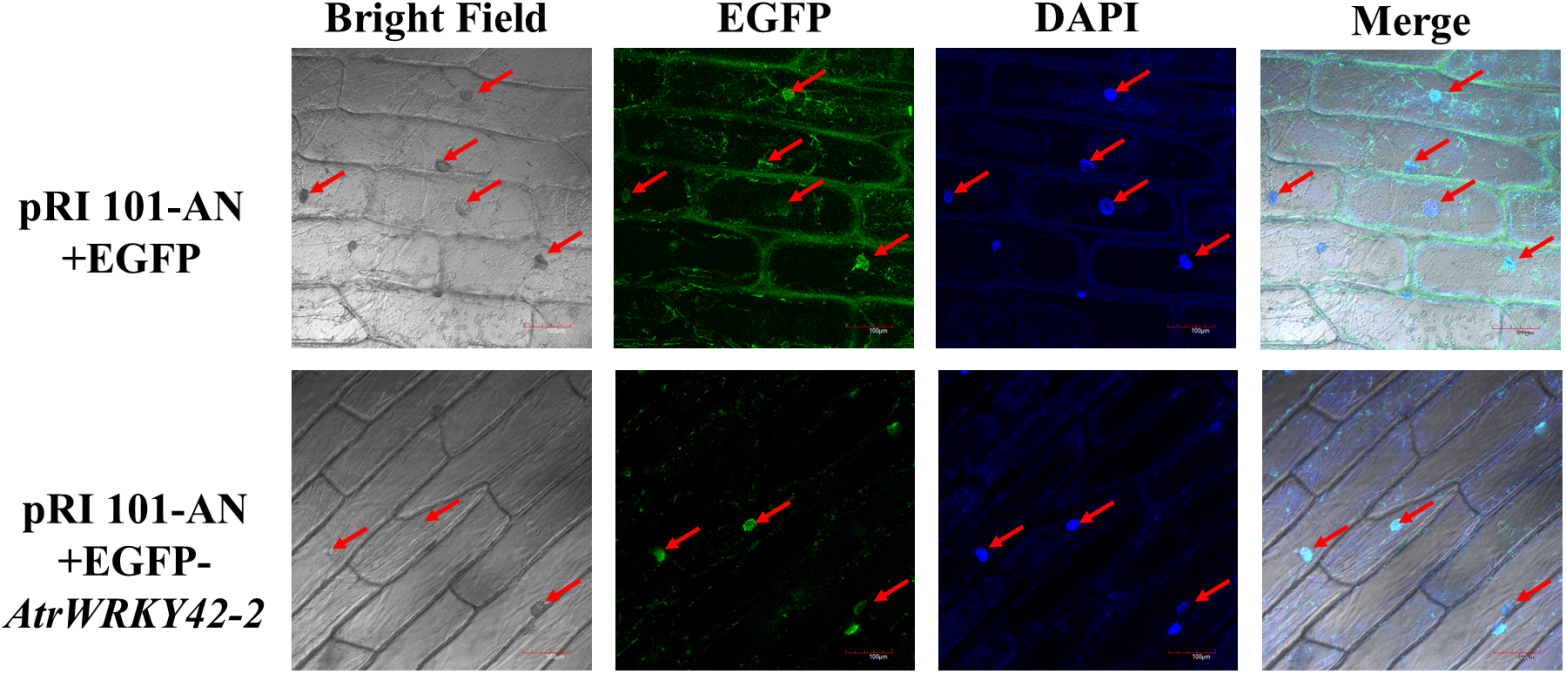
Subcellular localization of pRI 101-AN and *AtrWRKY42-2* proteins in onion. Red arrows indicate the location of EGFP and DAPI fluorescence signals in cells. Bars=100μm.

The full-length CDS of *AtrWRKY42-2* was fused with the EGFP gene to analyze its subcellular localization. Ater the transient expression of constructs in the inner epidermis of onion, green fluorescence of *AtrWRKY42-2* was detected exclusively in the nuclei (as evaluated by 4’,6-diamidino-2-phenylindole (DAPI) staining); however, in the positive control, the EGFP signal was observed around the cytoplasm, cell membrane, and nuclei (**Figure 7**).

### 3.6 Functional analysis of *AtrWRKY42-2*

#### 3.6.1 Overexpression of *AtrWRKY42-2* in amaranth

**Figure 8:**
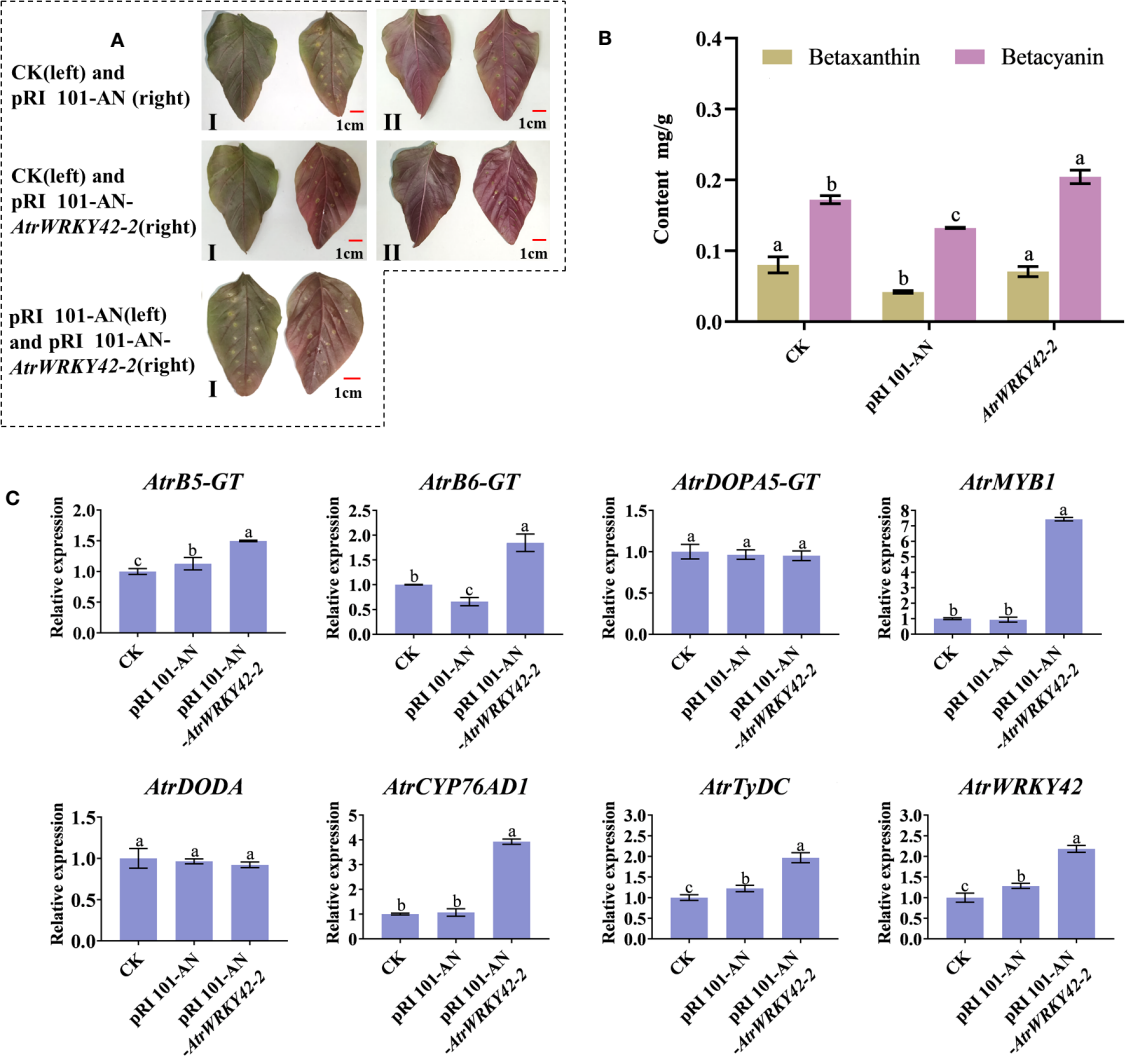
*Agrobacterium*-mediated transient transformation of the lower epidermis of amaranth leaves, revealing that the overexpression of *AtrWRKY42-2* promotes the synthesis of betalain in amaranth. **(A)** Plant leaves after transient transformation for 7 days (I: Blade face; II: Back of the blade) **(B)** Betalain content in leaves in plants with different transient transformations **(C)** Relative expression of betalain synthesis-related genes in leaves of plants with different transient transformations. (a, b and c indicate significant differences at p < 0.05; Bars: 1 cm).

As shown in **Figure 8A**, when compared with expression in the control and empty plasmid group, the overexpression of *AtrWRKY42-2* was visualized as dark red in the scales of ‘Suxian No.1’ amaranth. There were no significant differences in the betaxanthin content among transgenic plants, blank control group, and positive control group. However, the betacyanin content in transgenic plants was significantly higher (P < 0.05) than that in the control (**Figure 8B**). Results from the RT-qPCR analyses showed that *AtrWRKY42-2*, *CYP76AD1*, *TyDC*, *MYB1*, *B5-GT*, and *B6-GT* (Download ID and primer sequences are shown in **Supplementary Table S1**), key genes involved in betalain metabolism, were upregulated in plants overexpressing *AtrWRKY42-2* (**Figure 8C**). These findings were consistent with the leaf phenotype and betacyanin content.

#### 3.6.2 VIGS analysis of *AtrWRKY42-2*

**Figure 9:**
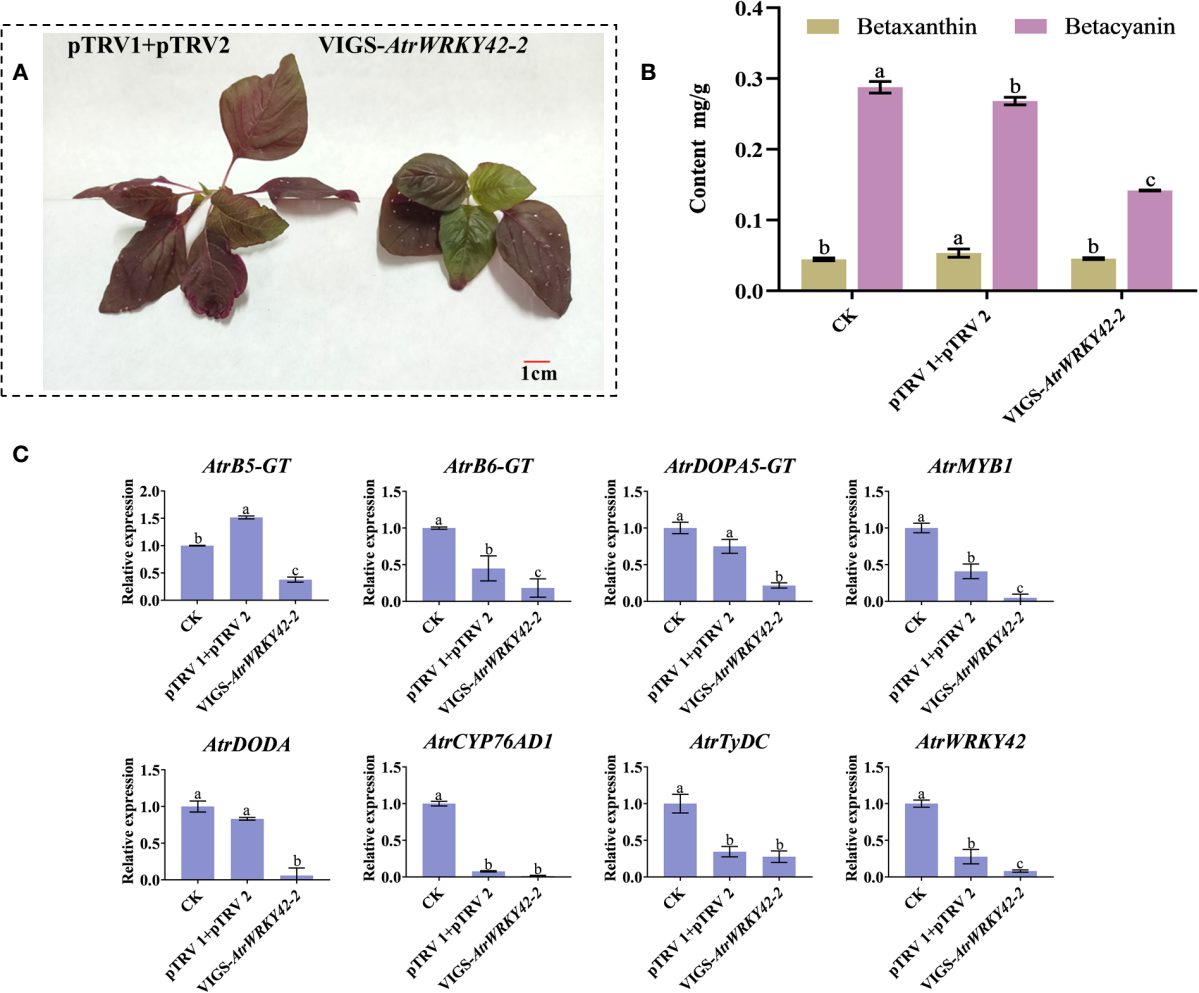
Silencing of *AtrWRKY42-2* inhibits betalain production. **(A)** Positive control plants (left) and VIGS transgenic positive plants (right) Phenotype. **(B)** Betalain content in leaves of positive control plants and VIGS transgenic plants. **(C)** Relative expression levels of key genes related to betalain synthesis in transgenic plants with gene silencing. Three biological replicates were performed for each sample (a, b and c indicate significant differences at p < 0.01; Bar = 1 cm).

The silencing of *AtrWRKY42-2* resulted in new green leaves (although not completely green) after 2–3 weeks of transformation (**Figure 9A**). There was no significant difference in the betaxanthin content between plants with gene silencing and the control; however, the betacyanin content in leaves was significantly lower in plants with gene silencing than in the control (**Figure 9B**). The RT-qPCR analysis showed that the expression levels of key genes in betalain synthesis were significantly decreased by gene silencing (**Figure 9C**). These results indicate that *AtrWRKY42-2* plays an important role in the betacyanin biosynthetic pathway of amaranth, and silencing *AtrWRKY42-2* inhibited the synthesis of betacyanin.

#### 3.6.3 Interaction of *AtrWRKY42-2* with the *AtrCYP76AD1* promoter

**Figure 10:**
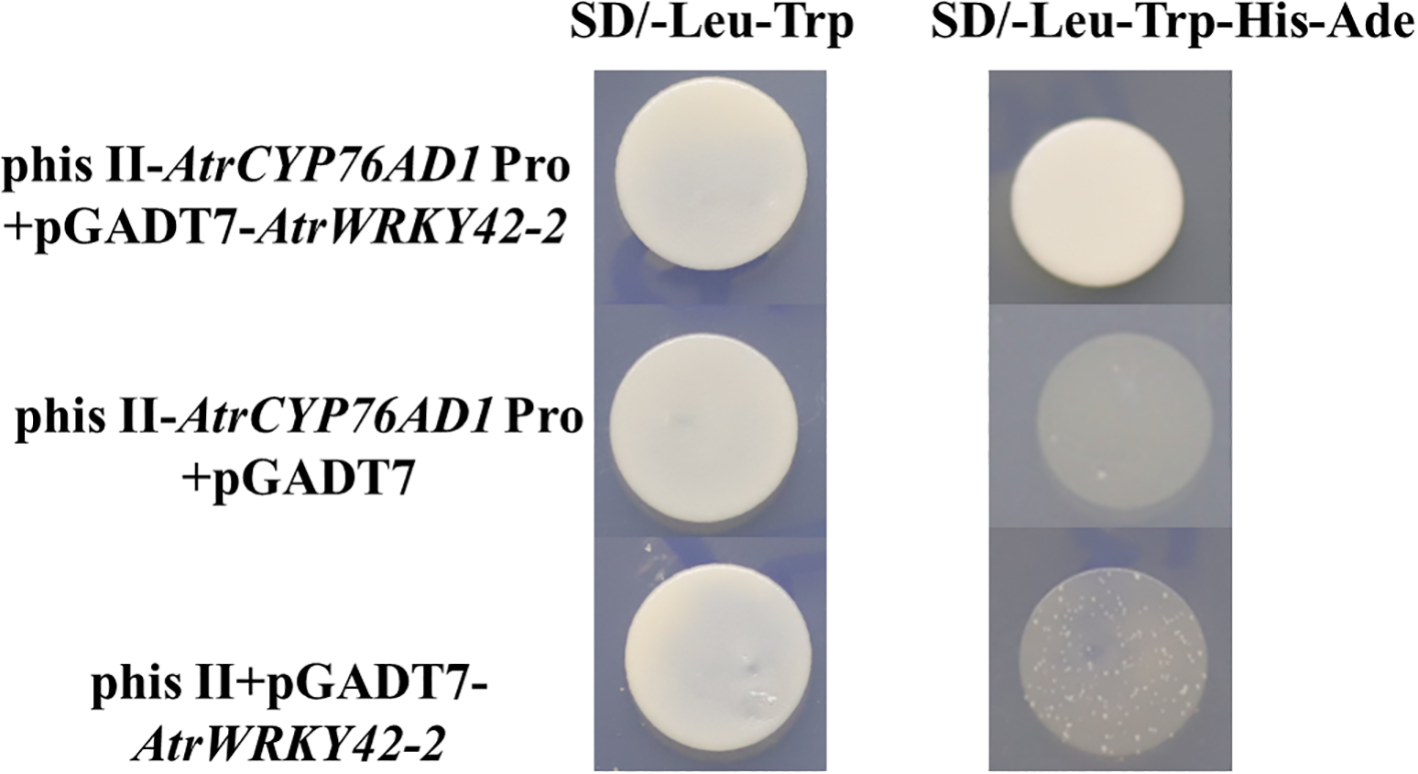
*AtrWRKY42-2* and *AtrCYP76AD1* promoter yeast one-hybrid assay. The experiment was repeated independently three times. SD/-Leu-Trp and SD/-Leu-Trp-His-Ade indicate yeast single defect medium.

The promoter of *AtrCYP76AD1* was cloned from ‘Suxian No.1’ amaranth (unpublished) (see **Supplementary Text S2** for the sequence). A typical W-box motif was identified in the *AtrCYP76AD1* promoter, which is a cognate binding site for WRKY TFs, suggesting that WRKY TFs are involved in the regulation of *AtrCYP76AD1*. Therefore, the interaction between *AtrWRKY42-2* and the promoter of *AtrCYP76AD1* was evaluated using a yeast one-hybrid assay. Yeast cells carrying only pHis II-AtrCYP76AD1-Pro could not grow on SD/-Leu-Trp-His-Ade medium (**Figure 10**). Yeast cells containing pHis II-AtrCYP76AD1-Pro+pGADT7-*AtrWRKY42-2* were grown on SD/-Leu-Trp-His-Ade medium (**Figure 10**). These results suggest that AtrWRKY42-2 binds directly to the promoter of *AtrCYP76AD1* and may be involved in the biosynthesis of amaranth betalains.

## 4 Discussion

The WRKY TFs are a universal gene family in higher plants, controlling plant growth and development under normal and stress conditions (Jiang et al., 2017; Gangola and Ramadoss, 2020; Li et al., 2020; Jia et al., 2022). However, *WRKY* gene family members in *A. tricolor* have not been identified and characterized. We screened 72 *AtrWRKY* genes from the *A. tricolor* transcriptome database, which was more than 70 genes reported in pitaya (Chen et al., 2022) and 2 genes in *Beta vulgaris* (Wu et al., 2019). AtrWRKYs were divided into four subfamilies according to their domain and zinc finger structure, consistent with the previous classification of WRKY TFs (Eulgem et al., 2000; Huang et al., 2012; Wang et al., 2014).

Betalains are a unique lineage in anthocyanin evolution (Alfonso et al., 2019; Li et al., 2019). However, they cannot coexist within the same plant. Betalains have only been detected in Caryophyllales species, with the exception of Caryophyllaceae and Molluginaceae species, as well as some higher fungi. The WRKY TFs has important roles in higher plants, including roles in plant growth and development and secondary metabolite synthesis under the normal and stress conditions (Peng et al., 2019; Hu et al., 2021; Chen et al., 2022). Recent studies have also shown that WRKY TFs are involved in betalain metabolism in pitaya (Gangola and Ramadoss, 2020; Hu et al., 2021; Chen et al., 2022). In pitaya, *HmoWRKY40* and *HpWRKY44* promote the synthesis of betalains (Cheng et al., 2017; Zhang et al., 2021), while *HmoWRKY42* inhibits the synthesis of betalains (Chen et al., 2022). A phylogenetic analysis revealed that *AtrWRKY42-2* was homologous to *HmoWRKY44* and *BvWRKY44*. However, the amino acid sequences of *BvWRKY44*, *AtrWRKY42-2*, and *HpWRKY44* in betalain-producing differed from *AtWRKY44* in *Arabidopsis* (**Figure 3C**). Some studies have found that *AtWRKY44* only regulates the biosynthesis of phenylpropyl and indole alkaloids (Johnson et al., 2002; Mao et al., 2011), no one studied the role of *AtWRKY44* in betalain synthesis, so we speculated that amino acid substitutions are involved in the regulation of betalain synthesis. Therefore, we speculated that the amino acid substitutions are involved in the regulation of betalain synthesis. Further studies are needed to verify whether the transfer of *AtWRKY44* into *A. thaliana* mediated by *A. tumefaciens* regulates anthocyanin synthesis and increases the anthocyanin content, similar to the other three WRKY homologues.

Betalain accumulation could make the amaranth leaves red (Barba-Espin et al., 2018; Fan et al., 2020), which is determined by environmental conditions and transcription factors (Erika et al., 2018; Preczenhak et al., 2019; Guerrero-Rubio et al., 2020; Sakuta et al., 2021; Sokolova et al., 2022). Our previous studies have indicated that various environmental factors and hormones (GA_3_ and 2,4-D) influence betalain biosynthesis (Xiao et al., 2021). The predicted cis-acting elements of *AtrWRKY42-2* contain TC-rich repeats, STREs, and so on based on a PlantCARE database analysis (**Supplementary Table S5**). Therefore, we speculated that these factors affect betalain synthesis via WRKY TFs. We found that the function of *AtrWRKY42-2* in amaranth is similar to those of *HpWRKY44* and *HmoWRKY40* in pitaya (Cheng et al., 2017; Zhang et al., 2021), and all of these loci promote the accumulation of betalains. When the WRKY domain and zinc finger structure of *AtrWRKY42-2* were silenced by VIGS technology, betacyanin synthesis was inhibited. In contrast, betacyanin accumulation in amaranth was promoted by *AtrWRKY42-2* overexpression. In analyses of the relative expression of *AtrWRKY42-2* and key genes involved in betalain synthesis, we observed that the gene expression levels were associated with the betacyanin content. These results indicated that *AtrWRKY42-2* promotes betacyanin synthesis. However, there was no difference in the betaxanthin content between transgenic plant and the control. These results content showed that *AtrWRKY42-2* was not involved in betaxanthin synthesis. Perhaps it was synthesis by other CYP76AD members and transcription factors. Previous studies have shown that the synthesis of betacyanin mainly depends on the action of *CYP76AD1* to synthesize cyclo-DOPA, and maintaining the ratio of *CYP76AD1* and CYP76ADβ (*CYP76AD5, 6, 15*) can regulate the synthesis ratio of betacyanin and betaxanthin. (Sunnadeniya et al., 2016). However, the regulation of *AtrWRKY42-2* in this study only led to the differential expression of key genes involved in the synthesis of betacyanin such as *CYP76AD1*, and it did not affect the expression of CYP76ADβ subfamily members (*CYP76AD5, 6, 15*) to exercise hydroxylation. Further functional studies of *AtrWRKY7* and *AtrWRKY40* are in progress.

**Figure 11:**
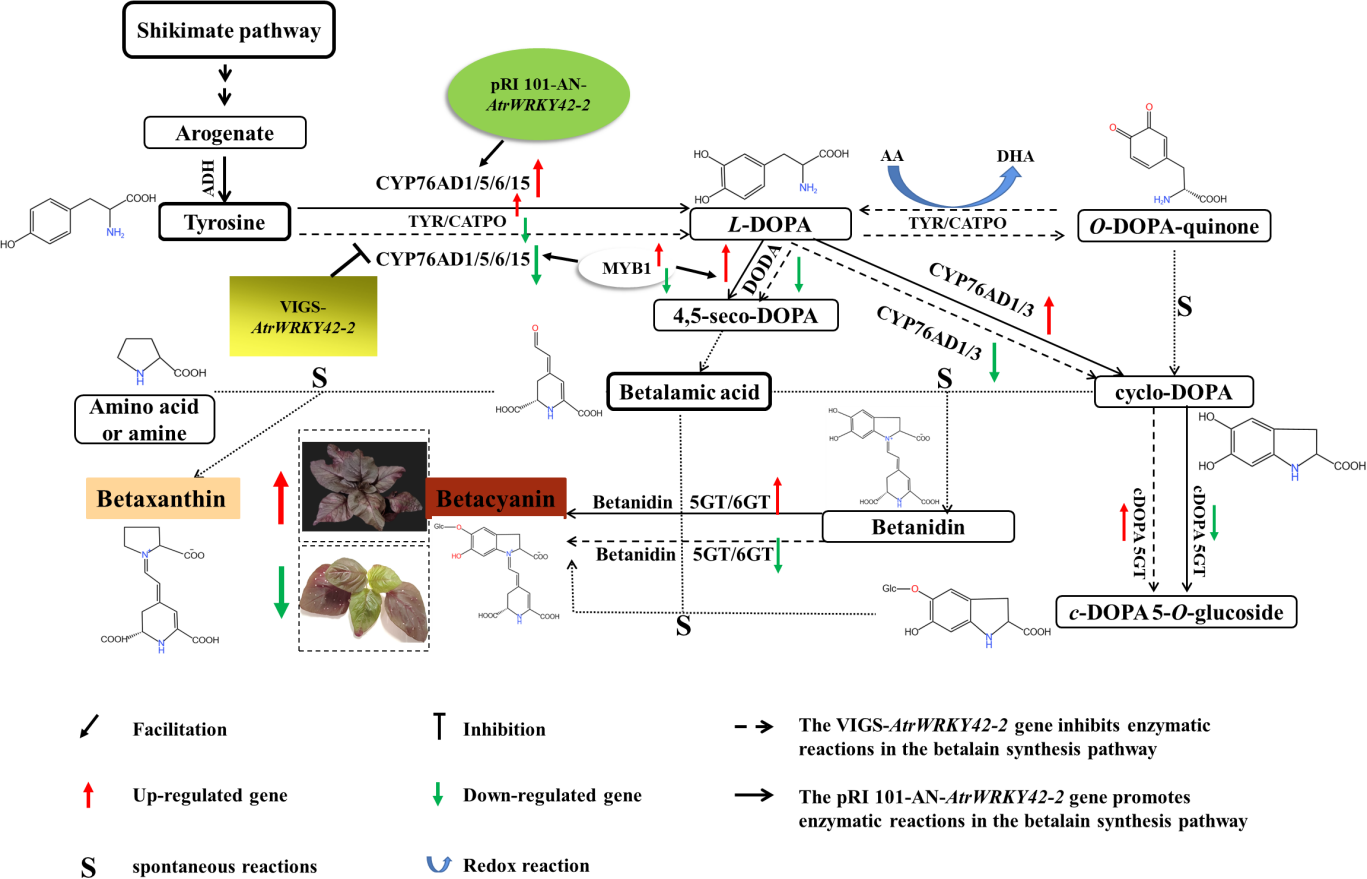
*AtrWRKY42-2* is involved in the regulation of betalain synthesis in ‘Suxian No.1’. In this study, Agrobacterium-mediated transient transformation of *AtrWRKY42-2* overexpression could promote the betalain synthesis and increase the content of betalain in ‘Suxian No.1’ amaranth. Moreover, the Agrobacteria-mediated virus-induced gene silencing system of *AtrWRKY42-2* could inhibit the synthesis of betalain in ‘Suxian No.1’ and reduce the content of betalain. The green and red arrows indicate down-regulation and up-regulation of key genes in the betalain synthesis pathway (*TyDC*, *CYP76AD1*, *DODA*, *DOPA5-GT*, *B5-GT*, *B6-GT*), respectively. AA, ascorbic acid; DHA, dehydroascorbate; GT, glucosyl transferase.

WRKY TFs recognize and bind to the TTGAC(C/T) W-box in the promoter regions of target genes (Chen et al., 2019). WRKY TFs can bind not only upstream promoters (Brand et al., 2013; Madhunita and Ralf, 2014) but also to upstream promoters of other genes regulated by WRKY TFs (Cheng et al., 2017; Li et al., 2020; Zhang et al., 2021; Chen et al., 2022; Zheng et al., 2022). The *AtrWRKY42-2* promoter sequence contained six W-box elements that specifically bind WRKY TFs. These results indicated that *AtrWRKY42-2* binds to itself or other WRKY TFs. In addition, the *AtrCYP76AD1* promoter sequence contains a W-box element of WRKY TFs. *AtrWRKY42-2* could activate *AtrCYP76AD1*, a key gene involved in betalain synthesis, by binding to its promoter responsible for betalain biosynthesis in amaranth. *HpWRKY44* and *HmoWRKY40* could activate *HmoCYP76AD1* expression by binding to its promoter responsible for betalain biosynthesis of pitaya (Cheng et al., 2017; Zhang et al., 2021). A yeast one-hybrid assay showed that *AtrWRKY42-2* interacts with the *AtrCYP76AD1* promoter (**Figure 10**), providing evidence that *AtrWRKY42-2* activates the transcription of *AtrCYP76AD1*, involved in amaranth betalain biosynthesis (**Figure 11**). *AtrWRKY42-2* transcript factor could regulate downstream gene transcription by binding W-box elements of target gene *AtrCYP76AD1* promoter, and then participate in betalain synthesis. The expression level of the key genes in the betalain synthesis pathway was up-regulated or down-regulated in accordance with overexpression or silencing expression of *AtrWRKY42-2* (**Figures 8**–**11**).

## 5 Conclusions

In summary, WRKY family members were screened using HMM in the amaranth transcriptome. A total of 72 WRKY family members were obtained, and these were divided into four groups according to the domain and zinc finger structure. These WRKY TFs were located in the nuclei, with the exception of *AtrWRKY50*. There were no significant differences in the expression levels of *AtrWRKY7\40\42* in the leaves of ‘Suxian No.1’, which may be explained by the complex factors involved in gene expression regulation, such that changes in any single factor do not cause substantial changes in expression. The expression level of *AtrWRKY42-2* in ‘Suamaranth 1’ was significantly higher than that of *AtrWRKY42-1*. A transient overexpression experiment and VIGS assay showed that *AtrWRKY42-2* promoted betalain synthesis. A yeast one-hybrid assay demonstrated that *AtrWRKY42-2* could bind to the *AtrCYP76AD1* promoter to regulate betalain synthesis.

## Author’s note

We declare that this manuscript is original, has not been published before, and is not currently being considered for publication elsewhere.

## Data availability statement

The datasets presented in this study can be found in online repositories. The names of the repository/repositories and accession number(s) can be found in the article/**Supplementary Material**.

## Author contributions

RY: Writing – original draft, Writing – review & editing. TH: Validation. WS: Conceptualization. ZA: Methodology. ZL: Supervision. SL: Writing – review & editing.

## References

[B1] Alfonso T. Tao F. Hester S. Nathanael W. -H. Samuel P. B. L. -N. Rui G.. (2019). The evolution of betalain biosynthesis in Caryophyllales. New Phytol. 224, 71–85. doi: 10.1111/nph.15980 31172524

[B2] Baillo E. H. Hanif M. S. Guo Y. Zhang Z. Algam S. A. (2020). Genome-wide Identification of WRKY transcription factor family members in sorghum (Sorghum bicolor (L.) moench). PloS One 15 (8), e0236651. doi: 10.1371/journal.pone.0236651 32804948 PMC7430707

[B3] Barba-Espin G. Glied-Olsen S. Dzhanfezova T. Joernsgaard B. Lütken H. Müller R. (2018). Preharvest application of ethephon and postharvest UV-B radiation improve quality traits of beetroot (Beta vulgaris L. ssp. vulgaris) as source of colourant. BMC Plant Biol. 18, 316. doi: 10.1186/s12870-018-1556-2 30509181 PMC6276243

[B4] Birkenbihl R. P. Kracher B. Ross A. Kramer K. Finkemeier I. Somssich I. E. (2018). Principles and characteristics of the Arabidopsis WRKY regulatory network during early MAMP-triggered immunity. Plant J. Cell Mol. Biol. 96 (3), 487-502. doi: 10.1111/tpj.14043 30044528

[B5] Brand L. H. Fischer N. M. Harter K. Kohlbacher O. Wanke D. (2013). Elucidating the evolutionary conserved DNA-binding specificities of WRKY transcription factors by molecular dynamics and *in vitro* binding assays. Nucleic Acids Res. 41 (21), 9764-9778. doi: 10.1093/nar/gkt732 23975197 PMC3834811

[B6] Cai Y. Xing J. Isun M. Corke H. (2006). Rapid identification of betacyanins from Amaranthus tricolor, Gomphrena globosa, and Hylocereus polyrhizus by matrix-assisted laser desorption/ionization quadrupole ion trap time-of-flight mass spectrometry (MALDI-QIT-TOF MS). J. Agric. Food Chem. 54 (18), 6520-6526. doi: 10.1021/JF0609983 16939305

[B7] Carreón-Hidalgo J. P. Román-Guerrero A. Navarro-Ocaña A. Gómez-Linton D. R. Franco-Vásquez D. C. Franco-Vásquez A. M.. (2022). Chemical characterization of yellow-orange and purple varieties of Opuntia ficus-indica fruits and thermal stability of their betalains. J. Food Sci. 88, 161-174. doi: 10.1111/1750-3841.16421 36524774

[B8] Chang Y. Chiu Y. Tsao N. Chou Y. Tan C. Chiang Y.. (2021). Author Correction: Elucidation of the core betalain biosynthesis pathway in Amaranthus tricolor. Sci. Rep. 11, 15634. doi: 10.1038/S41598-021-94665-9 34315918 PMC8316460

[B9] Chen C. Xie F. Kamran S. Hua Q. Chen J. Zhang Z.. (2022). Genome-wide identification of WRKY gene family in pitaya reveals the involvement of hmoWRKY42 in betalain biosynthesis. Int. J. Mol. Sci. 23 (18), 10568. doi: 10.3390/IJMS231810568 36142481 PMC9502481

[B10] Chen X. Li C. Wang H. Guo Z. (2019). WRKY transcription factors: evolution, binding, and action. Phytopathol. Res. 1, 13. doi: 10.1186/s42483-019-0022-x

[B11] Chen Z. Guo X. Chen Z. Chen W. Liu D. Zheng Y.. (2015). Genome-wide characterization of developmental stage- and tissue-specific transcription factors in wheat. BMC Genomics 16, 125. doi: 10.1186/s12864-015-1313-y 25766308 PMC4344791

[B12] Cheng M. (2018). Molecular mechanism of wrky transcription factor regulating betalain biosynthesis and sugar metabolism in pitaya fruit. doctorate. Dissertation, (South China Agricultural University). https://kns.cnki.net/KCMS/detail/detail.aspx?dbname=CMFD202002&filename=1018770423.nh

[B13] Cheng M. Huang Z. Hua Q. Shan W. Kuang J. Lu W.. (2017). The WRKY transcription factor HpWRKY44 regulates CytP450-like1 expression in red pitaya fruit (Hylocereus polyrhizus). Horticulture Res. 4, 17039. doi: 10.1038/hortres.2017.39 PMC5539414 28785415

[B14] Clement J. S. Mabry T. J. (1996). Pigment evolution in the caryophyllales: a systematic overview*. Botanica Acta 109 (5), 360–367. doi: 10.1111/j.1438-8677.1996.tb00584.x

[B15] Erika O. H. Jorge W. C. Jacobo-Velázquez D. A. (2018). Effects of UVB light, wounding stress, and storage time on the accumulation of betalains, phenolic compounds, and ascorbic acid in red prickly pear (opuntia ficus-indica cv. rojo vigor). Food Bioprocess Technol. 11 (12), 2265-2274. doi: 10.1007/s11947-018-2183-5

[B16] Eulgem T. Rushton P. J. Robatzek S. Somssich I. E. (2000). The WRKY superfamily of plant transcription factors. Trends Plant Sci. 5 (5), 199–206. doi: 10.1016/S1360-1385(00)01600-9 10785665

[B17] Fan R. Sun Q. Zeng J. Zhang X. (2020). Contribution of anthocyanin pathways to fruit flesh coloration in pitayas. BMC Plant Biol. 20, 361. doi: 10.1186/s12870-020-02566-2 32736527 PMC7394676

[B18] Gangola M. P. Ramadoss B. R. (2020). WRKY transcription factors for biotic and abiotic stress tolerance in plants. Transcription Factors Abiotic Stress Tolerance Plants, 15-28. doi: 10.1016/B978-0-12-819334-1.00002-2

[B19] Gins M. Gins V. K. Kononkov P. F. (2002). Change in the biochemical composition of amaranth leaves during selection for increased amaranthine content. Appl. Biochem. Microbiol. 38 (5), 474-479. doi: 10.1023/A:1019980821313 12391759

[B20] Guerrero-Rubio M. A. Hernández-García S. Escribano J. Jiménez-Atiénzar M. Cabanes J. García-Carmona F.. (2020). Betalain health-promoting effects after ingestion in Caenorhabditis elegans are mediated by DAF-16/FOXO and SKN-1/Nrf2 transcription factors. Food Chem. 330, 127228. doi: 10.1016/j.foodchem.2020.127228 32535316

[B21] Hatlestad G. J. Akhavan N. A. Sunnadeniya R. M. Elam L. Cargile S. Hembd A.. (2015). The beet Y locus encodes an anthocyanin MYB-like protein that activates the betalain red pigment pathway. Nat. Genet. 47, 92-96. doi: 10.1038/ng.3163 25436858

[B22] Hatlestad G. J. Sunnadeniya R. M. Akhavan N. A. Gonzalez A. Goldman I. L. Mcgrath J. M.. (2012). The beet R locus encodes a new cytochrome P450 required for red betalain production. Nat. Genet. 44 (7), 92-96. doi: 10.1038/ng.2297 22660548

[B23] Hu W. Ren Q. Chen Y. Xu G. Qian Y. (2021). Genome-wide identification and analysis of WRKY gene family in maize provide insights into regulatory network in response to abiotic stresses. BMC Plant Biol. 21, 427. doi: 10.1186/S12870-021-03206-Z 34544366 PMC8451115

[B24] Hua Q. Chen C. Chen Z. Chen P. Ma Y. Wu J.. (2016). Transcriptomic analysis reveals key genes related to betalain biosynthesis in pulp coloration of Hylocereus polyrhizus. Front. Plant Sci. 6, 1179. doi: 10.3389/fpls.2015.01179 26779215 PMC4700300

[B25] Huang S. Gao Y. Liu J. Peng X. Niu X. Fei Z.. (2012). Genome-wide analysis of WRKY transcription factors in Solanum lycopersicum. Mol. Genet. Genomics: MGG 287 (6), 495-513. doi: 10.1007/s00438-012-0696-6 22570076

[B26] Jia C. Wang Z. Wang J. Miao H. Zhang J. Xu B.. (2022). Genome-wide analysis of the banana WRKY transcription factor gene family closely related to fruit ripening and stress. Plants 11 (5), 662. doi: 10.3390/PLANTS11050662 35270130 PMC8912484

[B27] Jiang J. Ma S. Ye N. Jiang M. Cao J. Zhang J. (2017). WRKY transcription factors in plant responses to stresses. J. Integr. Plant Biol. 59 (2), 86–101. doi: 10.1111/jipb.12513 27995748

[B28] Johnson C. S. Kolevski B. Smyth D. R. (2002). TRANSPARENT TESTA GLABRA2, a trichome and seed coat development gene of Arabidopsis, encodes a WRKY transcription factor. Plant Cell 14 (6), 1359–1375. doi: 10.1105/tpc.001404 12084832 PMC150785

[B29] Li G. Meng X. Zhu M. Li Z. (2019). Research progress of betalain in response to adverse stresses and evolutionary relationship compared with anthocyanin. Molecules 24 (17), 3078. doi: 10.3390/molecules24173078 31450587 PMC6749444

[B31] Li W. Pang S. Lu Z. Jin B. (2020). Function and mechanism of WRKY transcription factors in abiotic stress responses of plants. Plants 9 (11), 1515. doi: 10.3390/plants9111515 33171689 PMC7695288

[B32] Liu S. Guo G. Wang W. X. Zhao C. Peng L. Zhang Z.. (2018). Effects of exogenous auxin substances and 6-BA on seed germination and seedling growth of Amaranth. Seed 37 (12), 92–96. doi: 10.16590/j.cnki.1001-4705.2018.12.092

[B33] Liu S. Zheng X. Pan J. Peng L. Cheng C. Wang X.. (2019). RNA-sequencing analysis reveals betalains metabolism in the leaf of Amaranthus tricolor L. PloS One 14 (4), e0216001. doi: 10.1371/journal.pone.0216001 31022263 PMC6483260

[B34] Madhunita B. Ralf O. (2014). WRKY transcription factors: Jack of many trades in plants. Plant Signaling Behav. 9 (2), e27700. doi: 10.4161/psb.27700 PMC4091213 24492469

[B35] Mao G. Meng X. Liu Y. Zheng Z. Chen Z. Zhang S. (2011). Phosphorylation of a WRKY transcription factor by two pathogen-responsive MAPKs drives phytoalexin biosynthesis in Arabidopsis. Plant Cell 23 (4), 1639–1653. doi: 10.2307/41433414 21498677 PMC3101563

[B36] Martins N. Roriz C. L. Morales P. Barros L. Ferreira I. C. F. R. (2017). Coloring attributes of betalains: a key emphasis on stability and future applications. Food Funct. 8 (4), 1357-1372. doi: 10.1039/c7fo00144d 28262892

[B37] Pan J. Peng L. Zhao C. Wang W. X. Zhang Z. Chen Y.. (2018). Cloning of plant amaARF6 and its response to exogenous hormones in Amaranthus seedlings. J. Northeast Agric. Univ. 49 (5), 24–32. doi: 10.19720/j.cnki.issn.1005-9369.2018.05.004

[B38] Parveen M. Ray S. Chatterjee N. C. (2018). Detection and diversity pattern of amaranth Cultivars originated in diverse region of Indian subcontinent. Int. J. Pharma Bio Sci. 9 (3). doi: 10.22376/ijpbs.2018.9.3.b66-71

[B39] Peng L. Wang Y. Sun X. Wang X. Liu S. (2019). Expression and functional analysis of AmMYB2 related to betalain metabolism of Amaranthus tricolor. Acta Hortic. Sin. 46 (3), 473-485. doi: 10.16420/j.issn.0513-353x.2018-0572

[B40] Polturak G. Aharoni A. (2018). “La Vie En Rose”: Biosynthesis, sources, and applications of betalain pigments. Mol. Plant 11 (1), 7-22. doi: 10.1016/j.molp.2017.10.008 29081360

[B41] Polturak G. Breitel D. Grossman N. Sarrion-Perdigones A. Weithorn E. Pliner M.. (2016). Elucidation of the first committed step in betalain biosynthesis enables the heterologous engineering of betalain pigments in plants. New Phytol. 210, 269-283. doi: 10.1111/nph.13796 26683006

[B42] Polturak G. Heinig U. Grossman N. Battat M. Leshkowitz D. Malitsky S.. (2018). Transcriptome and metabolic profiling provides insights into betalain biosynthesis and evolution in mirabilis jalapa. Mol. Plant 11, 189-204. doi: 10.1016/j.molp.2017.12.002 29247705

[B43] Preczenhak A. P. Orsi B. Lima G. P. P. Tezotto-Uliana J. V. Minatel I. O. Kluge R. A. (2019). Cysteine enhances the content of betalains and polyphenols in fresh-cut red beet. Food Chem. 286, 600-607. doi: 10.1016/j.foodchem.2019.02.040 30827652

[B44] Sakuta M. Tanaka A. Iwase K. Miyasaka M. Ichiki S. Hatai M.. (2021). Anthocyanin synthesis potential in betalain-producing Caryophyllales plants. J. Plant Res. 134 (6), 1335-1349. doi: 10.1007/S10265-021-01341-0 34477986 PMC8930957

[B45] Sheehan H. Feng T. Walker-Hale N. Lopez-Nieves S. Pucker B. Guo R.. (2020). Evolution of l-DOPA 4,5-dioxygenase activity allows for recurrent specialisation to betalain pigmentation in Caryophyllales. New Phytol. 227 (3), 914-929. doi: 10.1111/nph.16089 31369159 PMC7384185

[B46] Sokolova D. V. Shvachko N. A. Mikhailova A. S. Popov V. S. (2022). Betalain content and morphological characteristics of table beet accessions: their interplay with abiotic factors. Agronomy 12 (5), 1033. doi: 10.3390/AGRONOMY12051033

[B47] Strack D. Vogt T. Schliemann W. (2003). Recent advances in betalain research. Phytochemistry 62 (3), 247-269. doi: 10.1002/chin.200319225 12620337

[B48] Su M. Zuo W. Wang Y. Liu W. Zhang Z. Wang N.. (2022). The WKRY transcription factor MdWRKY75 regulates anthocyanins accumulation in apples (Malus domestica). Funct. Plant Biol. FPB 49 (9), 799-809. doi: 10.1071/FP21146 35577345

[B49] Sunnadeniya R. Bean A. Brown M. Akhavan N. Hatlestad G. Gonzalez A.. (2016). Tyrosine hydroxylation in betalain pigment biosynthesis is performed by cytochrome P450 enzymes in beets (Beta vulgaris). PloS One 11 (2), e0149417. doi: 10.1371/journal.pone.0149417 26890886 PMC4758722

[B50] Wang K. Liu Y. Song Z. Wang D. Qiu W.. (2019). Chelator complexes enhanced Amaranthus hypochondriacus L. phytoremediation efficiency in Cd-contaminated soils. Chemosphere 237, 124480. doi: 10.1016/j.chemosphere.2019.124480 31394449

[B51] Wang L. Zhu W. Fang L. Sun X. Su L. Liang Z.. (2014). Genome-wide identification of WRKY family genes and their response to cold stress in Vitis vinifera. BMC Plant Biol. 14, 103. doi: 10.1186/1471-2229-14-103 24755338 PMC4021059

[B52] Wu G. Li Z. Cao H. Wang J. (2019). Genome-wide identification and expression analysis of the WRKY genes in sugar beet ( Beta vulgaris L.) under alkaline stress. PeerJ 7, e7817. doi: 10.7717/peerj.7817 31632850 PMC6796966

[B53] Xiao F. Zheng Y. Chen J. Zhao C. Chen H. Wang L.. (2021). Selection and validation of reference genes in all-red Amaranth (;Amaranthus tricolor;L.) seedlings under different culture conditions. J. Hortic. Sci. Biotechnol. 96 (5), 604-613. doi: 10.1080/14620316.2021.1879686

[B54] Xie L. Liu S. Bai B. Y. Liu Y. Lin M. Cai S.. (2016). Cloning and expression analysis of betalain-related transcription factor gene amMYB1 in amaranthus tricolor L. Acta Botanica Boreali-Occidentalia Sin. 36 (6), 1080-1090. doi: 10.1007/s10529-015-2021-z

[B55] Zhang L. (2020). Transcription factors related to betalain biosynthesis in pitaya screening and functional analyses of MYB and WRKY. Master. Thesis, (South China Agricultural University).

[B56] Zhang L. Chen C. Xie F. Hua Q. Zhang Z. Zhang R.. (2021). A novel WRKY transcription factor HmoWRKY40 associated with betalain biosynthesis in pitaya (Hylocereus monacanthus) through regulating HmoCYP76AD1. Int. J. Mol. Sci. 22 (4), 2171. doi: 10.3390/IJMS22042171 33671670 PMC7926660

[B57] Zhang M. Chen Y. Nie L. Jin X. Liao W. Zhao S.. (2018). Transcriptome-wide identification and screening of WRKY factors involved in the regulation of taxol biosynthesis in Taxus chinensis. Sci. Rep. 8, 5197. doi: 10.1038/s41598-018-23558-1 29581461 PMC5980082

[B58] Zhao C. Fang X. Pan J. Peng L. Lai Z. Liu S. (2019). Cloning and expression analysis of atGAI gene in amaranthus tricolor L. Acta Botanica Boreali-Occidentalia Sin. 39 (2), 199-209. doi: 10.7606/j.issn.1000-4025.2019.02.0199

[B59] Zheng H. Hang Y. Wang X. Li X. Hu T. Tian Y.. (2022). Research progress of WRKY transcription factor family in medicinal plants. Plant Physiol. J. 58 (6), 1055–1067. doi: 10.13592/j.cnki.ppj.300030

[B60] Zheng X. Liu S. Cheng C. Guo R. Chen Y. Xie L.. (2016). Cloning and expression analysis of betalain biosynthesis genes in Amaranthus tricolor. Biotechnol. Lett. 38 (4), 723–729. doi: 10.1007/s10529-015-2021-z 26712368

