# Supplementary material for: Identification of WRKY gene family members in amaranth based on a transcriptome database and functional analysis of AtrWRKY42-2 in betalain metabolism
Source: Front Plant Sci. 2023 Dec 5;14:1300522. doi: 10.3389/fpls.2023.1300522 (PMC10734031; doi:10.3389/fpls.2023.1300522)
Supplement: Supplementary file 1 [file DataSheet_1.zip › Article supplement/Supplementary text S2.pdf]

附件 3：

Nucleotide sequences of *AtrWRKY42* promoter and *AtrCYP76AD1* promoter. W-box (T/C)TGAC(T/C) is indicated in box. TATA-box is shown in purple. Translation start site (ATG) is shown in red.

>Nucleotide sequence of AtrWRKY42-2 promoter

TAACCTGACTAGTTGTGGGTCTCACTGTTTTGGCAGAGTGCTTGTTGGTGGGTGCGCAGCATTATTGACCGTATAAAACCTAAATTT
CTATCTCAAAAAAATCTGTAATTTTTGGTCTATCCTCTTTCTTTTGTTACTAAATTGGATTTTATTGAACAATTTTTATTTTTAGTTGAGA
TATAGTCAAGGTCAAATCGTTCTTGGTAAATGAGGTTGTGGTAGAATAGTTTTGTAATTTATTGGGTTTGATATGCTCGGAGGCATAA
GCCAATGCTAACAATTACAAGTTACAACCCTTTTAGGCAGGATTTCCGGCACTGAGATGCTTGAACGAGTAAGCTAAGTGCGCTTCT
GAGCACTTACAACCCTTAGCATTTGCTTTGCTCGTGGTTGTGCGAATAGCCTAAGCTTAACCGAAATCACGGAAACAATAAAGCAAT
TAATCAATAATAAAGAGATTAAGACACAAAGGTTTTCTTATCATTGTGATCGAGTGATTGAGTCACCAGACCCCAAAATTCTTAATGA
CTTTTCTCAAAATGATCAACTCTAATTTTCTAAAACAAATTTATATAGTAAACCAAACAAAGAAACAGCTGCCAAAGATTGAGCCCT
CGAGGACTTGTTAAGCTCCTTCTCGACGACGACAACGAGTCATTGAGTCCGCATTTTTCCAGCCCTGTGAACTTCGTTTGTTCTGAA
ATTGAGCCTTCCCTTGCTTCCAAAATATTGGGAATCAAGTCATAACACGCCAATTAGTGATGGTTAACAGTTAACTCAGTTAACAATC
AATTTTAACTCTTATGCTCAACAGCTGATTCCTGGAGATGGAATTGGGTCTATAAAGTGTTTTAAAAGAAATAATAAGGTTATTATTAA
GGAACTGATTCACCTAAAGCTCAAATCGATGATTTTAGCCCCAGTAGCCCCAGGATATATTATATATTCTATCATGCCCCCTCACACAA
GAGTCTTTTGGGCTAGAGGTGCGGATGCATCTGTCACACGTGTTCAACCCGCTTACCAAAGAAGAAATAAGAAAAAGGGGACAGC
TGTCTTGTAATCATTAGTCATTAGATATTAAGTGCTTAGGTGTCTTAGGGGGTCTTGTGTTTGACTCCTATATAAGACTATCATTGTACA
TATTATGTTTTTGTCAAGTAAGAAATAAAAGGAAGTTTTGGGCAGGAGCTTAACAGTGTTCGAAACTGTGTTACCTTAGTGTGTGAA
TTAATATTTTCTTTCTTCCTTTAATCTCTATTTCTTGATAACTCAATTCCATAGCAATCTGGGCAGGGGACAGAACTGATCTGTCACAG
CATCATATGCCCTTTTCCTACATAGCACTAAATATTGCACTTGAAATAATGAGGGGTGGATGAGATTCAAACGATTTGAACCTATAATC
TTCCATTACGTTGATACGATGCTAGGAAACCAATTCAATCAAAAGCCTAGGTTGATGGTTGTAGCCTCAGTAGATTTTTTGTTCTGCT
TGTCGATTGTGAAGATGGTAGTTGTGTGCCCGATCAACAGTCAACTGGAAATAGCCATTCTACTATGACAGTGTAAGGCTGTGTATG
AATTCCTTACCCGATCGGAGAGATTTTTTAGGCATTGGAATAATGTTAATGTTTATGAGTCTATCATAAAAAAGCCATTGGAATAATGT
TGATAACTGCATTAGTGTTATTTTTCTATAAATCATCCATTCATTCTAGTCTTGCTCATTCATCTGTAGTTGACTTGTTGCAGCAGCATT
AACTGAATAAACTTTCTGAAGCAAGTCATTGATTATACAAACAAAAACGTTTCGTGTTCAGGAGGGTCAGGACTTAGTCTGATATTA
GAGCCTAATGAGCTGCTGTCACTTTGCTGTCTATTGATTGCA**ATG**

>Nucleotide sequence of AtrCYP76AD1 promoter

GGTCGACTGAGAAGAATTAATAGGTTGTATATAAGCCTAAATTATGTCCAAATAAATATTAAAATAGGTTAAGAACGAATTTATAATTGATTTTGGGTTGT
GTAAGTTGGGTCTAGATCAGATTTGGGTCTGGATTTAAATGGTACCTAAGAGAGAGTCTGGGGCGGTCTGAGTTTGAAGACCTTAGACCCAAAGCCTA
AAAATAGAGGTGGGCGTCTGATAGAGTCTTAGACCCATGATCATCTTTAAGTGTATGTCATTGCAAAATTAAAAAAAAAGGACAAATTCAATAGGATGA
AAATAAAGCTAATTACTTATGTACAAAGAATGATTGACACTATTGATGATTAATGATTATAATGTGGAAATAGCTTGTTTTAGGCTATCAAAGAAAAAATC
AGACAAAATGTATAATAGCAAATAACGTGGCCAAAATCAATGGTGTTGCTACTTGATACAATGCGACATGACTAACAGGTCATCTTGAGCCGAGCTATAT
TTGGGATTGCAACTGAATATTATCCTTCTATTTTTCAAATTTAATCACTTGAAAATGGCATGCTTGACGGGTCATGTCTAAGAACTTCGAGTTTGATCGAC
CTGCTTTTCATATTTGTAATTGTGACATGGCTAAATCGTTTATTCGAGTCATTAAGCTGATTTCGAATTAGATGTTTCGAGTCAGTTTGAAATCGAATTTTG
TATCTATATTGTTTTTATATAATCGTGAATTATATTTGAAATCGGGTTAAATCAGGTCCGATTACAAGCTTAGGTAAATTTCAGATTATAAGATCTCTTTTGA
ACACCTCTCACATGCTTGTAGCTTGAAAAATTTATTCATCATTCCCAACAATCATTCAAATATAAAAAGACATTATAGCGCTACAATAAATATATATGTTTA
ATCAAAATAAAAAAAATTAAATAATGTTGTGGATTCCAATCAAAAATATAAGGTGGAATTGAAAATTTTGATGTAGAACTGAAAATAGGTCAAGACGAT
CGAGTTTGAATTTCGATTTATTATAACCTAATTTTTTCCTTTTTAATTGGGTTATTTTTTGATCACGGGTCGTAATATTTATTAGTTATGACCCACTAAAACA
ATTTTGAAACGGGTTATAATCTGACAGTTTAAAATATAGCTAAGAGAGAATACATTTTAAAAATAATTGTGAGACCATCAAACCCCACTATAGATTTTCTA
TAATCAACTTATCAAAAATATCCGGTATCTACACACTGCAGTGTGCTCCAAATTTTTTTAACTTCATTTAACACTCAACAATCAACACATAGTCATACATT
GTCCATGCACTCACTACTCCTATATAAAAAGAGTAGATGAGCCTAAATAAGCACACAGCAACAGTTCTTTTTTCTATATATATCGCCTCATAACTTCTACC
TTATATATTATTTAAATGCTTTACTTTTTCCTTCTTATTTAAATTGTAGTACTATTTATTTTTGAAAAAAAAATTTTAAGTGTTTGTTTAATCACTTCTTC**ATG**
