# Supplementary material for: Identification of WRKY gene family members in amaranth based on a transcriptome database and functional analysis of AtrWRKY42-2 in betalain metabolism
Source: Front Plant Sci. 2023 Dec 5;14:1300522. doi: 10.3389/fpls.2023.1300522 (PMC10734031; doi:10.3389/fpls.2023.1300522)
Supplement: Supplementary file 1 [file DataSheet_1.zip › Article supplement/Supplementary text S4.pdf]

The gene protein sequence mentioned in Figure 4 of the article.
>AtrWRKY42-1
MEVKEIDRVIISKPVASRPNCTGFRLELLAGPINASLPPETAAAATRPKTVRPTTSQVKPTSE
IQVDTSEAAFDCSSTSISRVHNQSNIFKPLAKSVSKATISLLANLSSSGISHQQALSQFGVSA
KLDKLQIFSNQHSNNLSFVELSKSIEPPTIAPQMLDEELRMMASSTAGINKRSCDGYNWRK
YGQKQVKGSEFPRSYFKCTYPKCPMKKKVERSLDGQITEIVYKGEHNHPKPDHPKRNVS
QVQESAIAGTDQDHSISDPKHDNLCDEIEGFERQMENENSLMTSKQALFPGNYVPLFYNS
VDTEECSAGDRAIVDPAGIGRGCEEADQPTKVDDNGTKNKRRKQDKQNNDTGKSGEGVI
EPCILVHNNSEPEIMGDGFRWRKYGQKVVRRNPYPRSYYRCTSPKCNVRKHVERAMDDP
GTFITTYEGRHNHEMSMKTTNNSTTEPETQVLTIKDKQ
> AtrWRKY42-2
MEVKEIDRVIISKPVASRPNCTGFRLELLAGPINASLPPETAAAATRPKTVRPTTSQVKPTSE
IQVDTSEAAFDCSSTSISRVHNQSNIFKPLAKSVSKATISLLANLSSSGISHQQALSQFGVSA
KLDKLQIFSNQHSNNLSFVELSKSIEPPTIAPQMLDEELRMMASSTAGINKRSCDGYNWRK
YGQKQVKGSEFPRSYFKCTYPKCPMKKKVERSLDGQITEIVYKGEHNHPKPDHPKRNVS
QVQESAIAGTDQDHSISDPKHDNLCDEIEGFERQMENENSLMTSKQALFPGNYVPLFYNS
VDTEECSAGDRAIVDPAGIGRGCEEADQPTKVDDNGTKNKRR
>AtWRKY44
MEVNDGERVVIAKPVASRPSSSSGFRTFTELLTDSVTVSPQTTCHEIVDAAIRPKTLRFNQP
VAASVSCPRAEVKGIGNGMSCDDDSDSRNYVVYKPKAKLVSKATVSALANMLQGNRQQ
TWRQSEAVSYGKSVSQGTHRAGPNLVQKVPSFTESETSTGDRSSVDGYNWRKYGQKQV
KGSECPRSYYKCTHPKCPVKKKVERSVEGQVSEIVYQGEHNHSKPSCPLPRRASSSISSGF
QKPPKSIASEGSMGQDPNNNLYSPLWNNQSNDSTQNRTEKMSEGCVITPFEFAVPRSTNSN
PGTSDSGCKSSQCDEGELDDPSRSKRRKNEKQSSEAGVSQGSVESDSLEDGFRWRKYGQ
KVVGGNAYPRSYYRCTSANCRARKHVERASDDPRAFITTYEGKHNHHLLLSPPSSSTLPF
NSPQLSKQTI
>BvWRKY44
MEVKEMDRVVITKPIASRPNSTDFRMFSELLASPTPADTSSKMAVPAIRPKTVRVKSSISQA
PLAKSFQAGASGAAPYSSSDNMSRAQNQSTIYRPLAKVVSKATVSLLANMQGNSDIAHQ
QALSQADVSAQQDKHWLFSNQHPSNLSMVELSQSIEPPTISQQMLDGEPTMLTSSTASAD
RPSYDGYNWRKYGQKQVKGSEYPRSYYKCTHPNCLVKKKVERSLDGQITEIVYKGEHN
HPKPDHAKRSSSLSGVQELELPTAGPDQDLNSNAHKLCDDICDNNVGFERQMEIQNADPF
SKKSMLTEEFALSCNDHIESEKHNADDMIIEVSAGLGGGCEESVKGLKVHDDDGTKRKRR
KQENQNNAAGKSSKGVHEPQISVHNNTEPEIMGDGFRWRKYGQKVVKGNSYPRSYYRC
TNLKCNVRKHVERALDDPTTFITTYEGRHNHEMPVKMMNNSSAAEPET
> HpWRKY44
MLYLFRMELKDIDRVIIAKPVASRPICTGFRSFSELLAGAIDASPSSANLCSEMAVAAIRPKTI
RVKPAVNHSIVSQDEASRAASVSDKFARSENHSTMVFKPLAKVVSRATVSLLASMGDLNI
THPQAATQVDVGDQHLTQHKLFQVSNPCPSNPPQAEPSQVVDPPKMAQQMLDEEPKMIA
PSAANVDRPSYDGYNWRKYGQKQVKGSEYPRSYYKCTNPTCPVKKKVERSFDGQIAEIV
YKGEHNHPKPHPPKRNLSDGIGQGLHSTEPKLHESSTCARNEGFEGRIESQVGVGFSNPST
VPEKASVLCHHRTKIRKHNDGCATPEDSLKGDDNVHKSKRRKIDEHNEAGTSKEGIQEPRI
WVQNNTEPEISGDGFRWRKYGQKVVKGNPYPRSYYRCTGLKCNVRKHVERTSDDPAAFI
TTYEGRHNHEIPMKNTKNTTSPEPESQALTSKEK
